# Supplementary material for: Identification and impact of microbiota-derived metabolites in ascites of ovarian and gastrointestinal cancer
Source: Cancer Metab. 2025 May 13;13:21. doi: 10.1186/s40170-025-00391-5 (PMC12076955; doi:10.1186/s40170-025-00391-5)
Supplement: Supplementary file 1 — Supplementary Material 1 [file 40170_2025_391_MOESM1_ESM.docx]

**Additional Supplementary Information**

**Identification and impact of microbiota-derived metabolites in ascites of ovarian and gastrointestinal cancer**

Sisi Deng^1,2,3,4^, Wooyong Kim^3,4^, Kefan Cheng^3,4^, Qianlu Yang^1^, Yogesh Singh^5^, Gyuntae Bae^1,2^, Nicolas Bézière^1,7^, Lukas Mager^2,4,7,8,9^, Stefan Kommoss^6,^ Jannik Sprengel^3,4^, Christoph Trautwein^1,2,3,4^

^1^Department of Preclinical Imaging and Radiopharmacy, Werner Siemens Imaging Center, University Hospital Tübingen, Germany;

^2^Cluster of Excellence iFIT (EXC 2180) “Image Guided and Functionally Instructed Tumor Therapies”, University of Tübingen, Germany;

^3^Core Facility Metabolomics, Faculty of Medicine, University of Tübingen, Germany;

^4^M3 Research Center for Microbiome, Metabolome and Malignome, Medical Faculty of Tübingen, Germany;

^5^Institute of Medical Genetics and Applied Genomics, University of Tübingen, Germany;

^6^Diak Klinikum, Department of Obstetrics and Gynecology, Schäbisch Hall, Germany ;

^7^Cluster of Excellence CMFI (EXC 2124) “Controlling Microbes to Fight Infections”, Eberhard Karls University of Tübingen, Tübingen, Germany;

^8^Department of Internal Medicine I, Faculty of Medicine, University of Tübingen, Germany;

^9^Department of Physiology and Pharmacology, Snyder Institute for Chronic Diseases, Cumming School of Medicine, University of Calgary, Calgary, AB, T2N 4N1, Canada

* Authors to whom correspondence should be addressed

[christoph.trautwein@med.uni-tuebingen.de](mailto:christoph.trautwein@med.uni-tuebingen.de)

The authors declare no potential conflicts of interest.

**Table S1.** Cytokine/Chemokine values Changes Between OC and GI

| Cytokine/Chemokine | V | P.Value | LOG10(p) | FDR |
| --- | --- | --- | --- | --- |
| IL-10 | 6.5 | 0.00335 | 2.4749 | 0.033501 |
| IFN-α2 | 19.5 | 0.07541 | 1.1226 | 0.37705 |
| IL-1β | 57.5 | 0.13072 | 0.88366 | 0.43573 |
| MCP-1 | 54 | 0.23699 | 0.62528 | 0.59246 |
| IL-6 | 28 | 0.31542 | 0.50112 | 0.63083 |
| IL-8 | 49 | 0.45976 | 0.33747 | 0.66719 |
| TNF-α | 31.5 | 0.47697 | 0.32151 | 0.66719 |
| IL-23 | 32.5 | 0.53375 | 0.27266 | 0.66719 |
| IL-18 | 45 | 0.69647 | 0.1571 | 0.77385 |
| IFN-γ | 38.5 | 0.92916 | 0.031908 | 0.92916 |

**Table S2.** Cytokine/Chemokine values Changes Between OC II-III and OC IV Groups

| Cytokine/Chemokine | V | P.Value | LOG10(p) | FDR |
| --- | --- | --- | --- | --- |
| IL6 | 3 | 0.11667 | 0.93305 | 1 |
| MCP1 | 6 | 0.38333 | 0.41642 | 1 |
| IL23 | 6 | 0.38333 | 0.41642 | 1 |
| IFNr | 7 | 0.51667 | 0.28679 | 1 |
| IL1B | 8 | 0.66667 | 0.17609 | 1 |
| TNFa | 9 | 0.83333 | 0.079181 | 1 |
| IL10 | 12 | 0.83333 | 0.079181 | 1 |
| IFNa2 | 10 | 1 | 4.82E-17 | 1 |
| IL8 | 11 | 1 | 0 | 1 |
| IL18 | 11 | 1 | 0 | 1 |

**Table S3** List of all annotated metabolites with annotation quality (AQ) scores derived from Metaboscape.

| Name | AQ | MS/MS |
| --- | --- | --- |
| (.+/-.)-N,N-Dimethyl-3,4-methylenedioxyamphetamine | 20112 | TRUE |
| (+)-(S)-Carvone | 20202 | TRUE |
| (+)-11-Nor-.DELTA.9-tetrahydrocannabinol-9-carboxylic acid glucuronide | 20110 | TRUE |
| (±)9-HpODE | 20210 | TRUE |
| (±)-Hexanoylcarnitine | 20222 | TRUE |
| (1R,4AR)-6-Hydroxy-1,4a-dimethyl-7-propan-2-yl-2,3,4,4b,5,6,10,10a-octahydrophenanthrene-1-carboxylic acid | 20201 | TRUE |
| (1S,4R)-Bicyclo[2.2.1]hept-2-ylmethanamine | 20200 | FALSE |
| (2E)-2,5-Dimethyl-4-vinyl-2,5-hexadien-1-yl ?-D-glucopyranoside | 20200 | FALSE |
| (2E)-4-Oxo-4-[2-(3-pyridinylcarbonyl)hydrazino]-2-butenoic acid | 20200 | FALSE |
| (2-methoxypyrimidin-5-yl)-pyrrolidin-1-ylmethanone | 20200 | FALSE |
| (2R)-3-Hydroxyisovaleroylcarnitine | 10222 | TRUE |
| (2S,4S)-1-cyclobutyl-4-phenoxypyrrolidine-2-carboxamide | 20200 | FALSE |
| (3.beta.)-Allopregnanolone sulfate | 20220 | TRUE |
| (3-Methoxy-4-hydroxyphenyl)ethylene glycol sulfate | 20120 | TRUE |
| (4-Methylphenyl)oxidanesulfonic acid | 20210 | TRUE |
| (5AR,10aR)-Octahydrodipyrrolo[1,2-a:1',2'-D]pyrazine-5,10-dione | 10220 | TRUE |
| (5E,9E)-Farnesylacetone | 20200 | FALSE |
| (9Z)-5,8,11-Trihydroxyoctadec-9-enoic acid | 20210 | TRUE |
| (9Z,12E)-15,16-Dihydroxyoctadeca-9,12-dienoic acid | 20110 | TRUE |
| (9Z,12Z)-15-Hydroxy-16-methoxyoctadeca-9,12-dienoic acid | 20200 | FALSE |
| (CIS-) NANOPHINE | 20200 | FALSE |
| (E)-2-(Hydroxymethyl)-3-(3-oxo-5-propan-2-yl-4,5,6,7-tetrahydro-1H-2-benzofuran-4-yl)prop-2-enoic acid | 20200 | FALSE |
| (E)-Ethyl 3-(2-cyanophenyl)acrylate | 10210 | TRUE |
| (R)-3-Hydroxybutyric acid | 20200 | FALSE |
| (R)-Butyrylcarnitine | 10222 | TRUE |
| .alpha.-Hydroxymetoprolol | 10212 | TRUE |
| .alpha.-Methylcinnamic acid | 20200 | FALSE |
| .alpha.-Tocotrienol | 20110 | TRUE |
| [3-[4-(cyclopropylmethoxymethyl)triazol-1-yl]azetidin-1-yl]-(6-methoxypyridin-3-yl)methanone | 20200 | FALSE |
| [3-[4-(trifluoromethoxy)phenyl]-1,2,4-oxadiazol-5-yl]methyl 2,5-dimethyl-1,1-dioxo-1,2,6-thiadiazine-4-carboxylate | 20200 | FALSE |
| [7-(2-Hydroxypropan-2-yl)-4a-methyl-1-methylidene-2,3,4,5,6,7,8,8a-octahydronaphthalen-2-yl] acetate | 20200 | FALSE |
| [8]-Dehydrogingerdione | 20200 | FALSE |
| 1- (2-METHOXYPHENYL)PIERAZINE | 20222 | TRUE |
| 1-(1',3'-Benzodioxol-5'-yl)-2-butanamine | 20112 | TRUE |
| 1-(1Z-Hexadecenyl)-sn-glycero-3-phosphocholine | 20220 | TRUE |
| 1-(2-Hydroxyethyl)-2,2,6,6-tetramethyl-4-piperidinol | 20222 | TRUE |
| 1-(p-Tolyl)cyclopropanecarboxylic acid | 10222 | TRUE |
| 1,2-Bis(O-decanoyl)-sn-glyceryl-3-phosphorylcholine | 10102 | FALSE |
| 1,2-Dilinoleoylglycerol | 10210 | TRUE |
| 1,2-Dimethylimidazole | 20200 | FALSE |
| 1,3-Bis(2-methoxyphenyl)thiourea | 20102 | FALSE |
| 1,3-Dimethyluracil | 20200 | TRUE |
| 1,3-Dimethyluric acid | 20110 | TRUE |
| 1,4-Dimethyl-2,6-dioxo-1,2,5,6-tetrahydropyridine-3-carbonitrile | 20200 | FALSE |
| 1,8-Diazabicyclo[5.4.0]undec-7-ene | 20220 | TRUE |
| 1.alpha.-Methyl-5.alpha.-androstan-3.alpha.-ol-17-one glucuronide | 20111 | TRUE |
| 10-Hydroxy-2-decenoic acid | 20210 | TRUE |
| 10-Hydroxydecanoic acid | 20220 | TRUE |
| 11,14,17-Eicosatrienoic acid, (Z,Z,Z)- | 20200 | FALSE |
| 11.alpha.-Hydroxyprogesterone .beta.-D-glucuronide | 20111 | TRUE |
| 11a-Hydroxyprogesterone | 20101 | TRUE |
| 11-Hydroxyundecanoic acid | 20020 | TRUE |
| 12(13)-Epoxy-9Z-octadecenoic acid | 20210 | TRUE |
| 12-Hydroperoxy-5Z,8Z,10E,14Z,17Z-eicosapentaenoic acid | 20110 | TRUE |
| 12-Hydroxydodecanoic acid | 20201 | FALSE |
| 13E-Docosenamide | 20220 | TRUE |
| 13-Hydroxy-9Z,11E-octadecadienoic acid | 20220 | TRUE |
| 13-Keto-9Z,11E-octadecadienoic acid | 20210 | TRUE |
| 13S-HOTrE(gamma) | 20210 | TRUE |
| 13S-Hydroxy-9Z,11E,15Z-octadecatrienoic acid | 20210 | TRUE |
| 13Z-Docosenamide | 10220 | TRUE |
| 14,15-EE-5(Z)-E | 20210 | TRUE |
| 14,15-Epoxy-5Z,8Z,11Z-eicosatrienoic acid | 20120 | TRUE |
| 14-Hydroxymyristic acid | 20111 | TRUE |
| 15(S)-HETrE | 20120 | TRUE |
| 15(S)-Hydroxy-(5Z,8Z,11Z,13E)-eicosatetraenoic acid | 20220 | TRUE |
| 15S-HEPE | 20220 | TRUE |
| 16,16-Dimethylprostaglandin A1 | 20110 | TRUE |
| 16-Hydroxyhexadecanoic acid | 20222 | TRUE |
| 17.beta.-Hydroxy-17.alpha.-methyl-5.alpha.-androstan-1-en-3-one | 10122 | TRUE |
| 17-Acetoxygrindelic acid | 20101 | FALSE |
| 17a-Estradiol | 20202 | FALSE |
| 17-Epioxandrolone | 10111 | TRUE |
| 17-Hydroxy-4Z,7Z,10Z,13Z,15E,19Z-docosahexaenoic acid | 20210 | TRUE |
| 17-Trifluoromethylphenyl-13,14-dihydrotrinorprostaglandin F1.alpha. | 10102 | FALSE |
| 18,19-Dihydroxy-3-cleroden-15-oic acid | 20102 | FALSE |
| 1-Decanoyl-2-hydroxy-sn-glycero-3-phosphocholine | 20220 | TRUE |
| 1-Dodecanamine | 10220 | TRUE |
| 1-Dodecyl-2-pyrrolidinone | 20200 | FALSE |
| 1-ethyl-N-methylindazole-6-carboxamide | 20200 | FALSE |
| 1-ethyl-N-phenylpyrazole-3-carboxamide | 10210 | TRUE |
| 1-Formylpyrrolidine-2-carboxylic acid | 20200 | FALSE |
| 1'-Hydroxymidazolam .beta.-D-glucuronide | 10112 | TRUE |
| 1-Isopropyl-3-methylbenzene | 20200 | FALSE |
| 1-Lignoceroyl-2-hydroxy-sn-glycero-3-phosphocholine | 20222 | TRUE |
| 1-Methyl-4-nitro-1H-imidazole | 10222 | TRUE |
| 1-Methyladenosine | 20102 | TRUE |
| 1-Methylguanine | 20202 | TRUE |
| 1-Methylhistidine | 20102 | TRUE |
| 1-O-Octadecyl-sn-glyceryl-3-phosphorylcholine | 20220 | TRUE |
| 1-O-Palmitoyl-2-O-acetyl-sn-glycero-3-phosphorylcholine | 20112 | TRUE |
| 1-Phenylethylamine | 20201 | TRUE |
| 1-Piperazineethanol, 4-dibenzo[b,f][1,4]thiazepin-11-yl- | 10122 | TRUE |
| 2-(1H-Imidazol-1-yl)-1-phenylethanone | 10210 | TRUE |
| 2-(1-Methylbutyl)phenol | 20200 | FALSE |
| 2-(2-Butoxyethoxy)acetic acid | 20200 | FALSE |
| 2-(4-Fluorophenyl)-N-(2-nitrobenzyl)ethanamine | 20101 | FALSE |
| 2-(4-methylanilino)pyridine-3-sulfonamide | 10220 | TRUE |
| 2-(5-phenyl-1,2,4-oxadiazol-3-yl)ethanamine | 20200 | FALSE |
| 2-(5-pyrazin-2-yl-1,3,4-oxadiazol-2-yl)-N-[3-(trifluoromethyl)phenyl]acetamide | 20200 | FALSE |
| 2-(Hydroxymethyl)-6-methylpyridin-3-ol | 20200 | FALSE |
| 2-(hydroxymethyl)-Pyrimidine | 20200 | FALSE |
| 2-(N-Ethyl-N-m-toluidino)ethanol | 10222 | TRUE |
| 2,2'-[(4-Methylphenyl)imino]diethanol | 10221 | TRUE |
| 2,2'-Methylenebis(ethyl-6-tert-butylphenol) | 20220 | TRUE |
| 2,3,4,5-Tetrahydro-1H-2-benzazepine | 20110 | TRUE |
| 2,3-Diaminonaphthalene | 20200 | FALSE |
| 2,4,5-Trimethoxydihydrocinnamic acid | 20201 | FALSE |
| 2,4-Diisopropylphenol | 20002 | FALSE |
| 2,4-Dimethylthiazole-5-carboxylic acid | 20200 | FALSE |
| 2,4-Dodecadienoic acid isobutylamide | 10212 | TRUE |
| 2,5,7,8-Tetramethyl-2-(.beta.-carboxyethyl)-6-hydroxychroman | 20210 | TRUE |
| 2,5,8,11,14-Pentaoxahexadecan-16-ol | 10220 | TRUE |
| 2,6-Dimethylpyrazine | 20210 | TRUE |
| 2.alpha.-Methyl-5.alpha.-androstan-3.alpha.-ol-17-one | 20202 | FALSE |
| 2-[(3-morpholin-4-yl-7-azaspiro[3.5]nonan-7-yl)methyl]benzonitrile | 20200 | FALSE |
| 2-[(7-Amino-7H-[1,2,4]triazolo[4,3-b][1,2,4]triazol-3-yl)sulfanyl]-N-(2-pyrazinyl)acetamide | 10102 | FALSE |
| 2-[4-(4-methylphenyl)-9-propan-2-yl-1,5,9-triazaspiro[5.5]undec-4-en-2-yl]phenol | 20200 | FALSE |
| 2-[4-(cyclopropylmethyl)-1,2,4-triazol-3-yl]-1-[(3,4-difluorophenyl)methyl]piperidine | 20200 | FALSE |
| 2-[8-[cyclohexyl(methyl)amino]-3-oxo-[1,2,4]triazolo[4,3-a]pyrazin-2-yl]-N-(3-ethylphenyl)acetamide | 20110 | TRUE |
| 21-hydroxy-heneicosanoic acid | 20102 | FALSE |
| 2-Acetamidooctanoic acid | 20021 | TRUE |
| 2-Amino-2-methyl-4-phenylbutanoic acid | 20210 | TRUE |
| 2-Amino-5-methylhexanoic acid | 20220 | TRUE |
| 2'-Aminoacetophenone | 20200 | FALSE |
| 2-Aminonaphthalene | 10211 | TRUE |
| 2-Cyano-N-(prop-2-en-1-yl)acetamide | 20200 | FALSE |
| 2-Cyclohexylamino-2-oxazoline | 20200 | FALSE |
| 2-ethoxy-1-[4-[4-methyl-5-(4-methylphenyl)-1,1-dioxo-1,2-thiazol-3-yl]-1,4-diazepan-1-yl]ethanone | 20110 | TRUE |
| 2-Ethoxy-5-(1-propenyl)phenol | 20200 | FALSE |
| 2-Ethyl-4-methyl-1H-imidazole | 20011 | TRUE |
| 2-Ethylhexyl dihydrogen phosphate | 20221 | TRUE |
| 2-Hydroxy-3-isopropyl-6-methylbenzoic acid | 10120 | TRUE |
| 2-Hydroxy-3-methylbutyric acid | 20221 | TRUE |
| 2-Hydroxycaproic acid | 20200 | TRUE |
| 2-Hydroxymyristic Acid | 20221 | TRUE |
| 2-Hydroxypalmitic acid | 10221 | TRUE |
| 2-Hydroxyphenethylamine | 20201 | TRUE |
| 2-Methoxyestradiol | 20201 | TRUE |
| 2-Methyl-1-Pyrroline | 20200 | FALSE |
| 2-Methyl-3-ketovaleric acid | 20200 | TRUE |
| 2-Methylcinchoninamide | 10212 | TRUE |
| 2-Methylguanosine | 20220 | TRUE |
| 2-Methylindoline | 10210 | TRUE |
| 2-Phenylbutyric acid | 20101 | TRUE |
| 2-Phenylglycine | 20201 | TRUE |
| 2-Pyrocatechuic acid | 20221 | TRUE |
| 2-Pyrrolidinone | 20200 | FALSE |
| 2-tert-Butyl-4-ethylphenol | 20212 | TRUE |
| 2-Thenoylglycine | 20200 | FALSE |
| 3-(2,5,7-trimethylpyrazolo[1,5-a]pyrimidin-6-yl)propanoic acid | 20200 | FALSE |
| 3-(2-methoxyphenyl)-5,7-dimethylpyrazolo[1,5-a]pyrimidine | 10210 | TRUE |
| 3-(2-Methoxyphenyl)propanoic acid | 20221 | TRUE |
| 3-(2-Oxocyclohexyl)propanoic acid | 20210 | TRUE |
| 3-(4-Isopropoxyphenyl)propanoic acid | 20221 | TRUE |
| 3-(4-methylphenyl)-5-[2-(4-pyrimidin-2-ylpiperazin-1-yl)sulfonylphenyl]-1,2,4-oxadiazole | 20200 | FALSE |
| 3-(4-Phenyl-5-sulfanyl-4H-1,2,4-triazol-3-yl)-1-propanol | 20202 | FALSE |
| 3-(Cyclohexylamino)-2-hydroxy-1-propanesulfonic acid | 20120 | TRUE |
| 3,19-Dihydroxyurs-12-ene-23,28-dioic acid | 10102 | FALSE |
| 3,4,5-Trimethoxycinnamic acid | 20101 | TRUE |
| 3,4-Dihydroxyhydrocinnamic acid | 20200 | TRUE |
| 3,4-Dimethoxymethcathinone | 20210 | TRUE |
| 3,4-Dimethyl-1,2-cyclopentadione | 20021 | TRUE |
| 3',4'-Methylenedioxy-.alpha.-pyrrolidinopropiophenone | 20210 | TRUE |
| 3,5-Dimethoxyphenol | 20200 | FALSE |
| 3,5-Dimethylmorpholine | 20220 | TRUE |
| 3,7-Dimethyl-2,6-octadienenitrile | 20210 | TRUE |
| 3,7-Dimethyluric acid | 20220 | TRUE |
| 3-[(5,6-Diphenylfuro[2,3-D]pyrimidin-4-yl)amino]-1-propanol | 20102 | FALSE |
| 3-[1-[6-(ethylamino)pyrimidin-4-yl]piperidin-3-yl]-N-[2-(4-methylpiperazin-1-yl)ethyl]propanamide | 20200 | FALSE |
| 3-[5-(methoxymethyl)-1,2,4-oxadiazol-3-yl]-N-(2-methylpropyl)pyrrolidine-1-carboxamide | 10210 | TRUE |
| 3-Acetyl-7-diethylaminocoumarin | 10102 | FALSE |
| 3-Amino-4-ethylbenzenesulfonic acid | 20220 | TRUE |
| 3-Aminononanoic acid | 20200 | FALSE |
| 3-Aminopentan-2-ol | 20200 | FALSE |
| 3b-Hydroxy-5-cholenoic acid | 20101 | FALSE |
| 3-Cysteinylacetaminophen | 10220 | TRUE |
| 3-Dehydroepiandrosterone sulfate | 20120 | TRUE |
| 3-Dimethylaminopropionitrile | 20200 | FALSE |
| 3-Hexanone | 20200 | TRUE |
| 3-Hexenedioic acid | 20200 | TRUE |
| 3-Hydroxy-3-(nitromethyl)-1,3-dihydro-2H-indol-2-one | 20200 | FALSE |
| 3-Hydroxyanthranilic acid | 20201 | TRUE |
| 3-Hydroxybutyrylcarnitine | 20221 | TRUE |
| 3-Hydroxycapric acid | 20220 | TRUE |
| 3-Hydroxyhexadecanoylcarnitine | 20120 | TRUE |
| 3-Hydroxyisovaleric acid | 20201 | TRUE |
| 3-Hydroxyoleylcarnitine | 10220 | TRUE |
| 3'-Hydroxyropivacaine | 10221 | TRUE |
| 3-Indolepropionic acid | 20201 | TRUE |
| 3-Indolepropionic acid | 20201 | TRUE |
| 3-Methoxy-4-(2-methylpropoxy)benzoic acid | 20211 | TRUE |
| 3-Methoxycatechol | 20200 | FALSE |
| 3-Methoxyphenylacetic acid | 20200 | TRUE |
| 3-methyl-1-(2-phenyl-7,8-dihydro-5H-pyrido[4,3-d]pyrimidin-6-yl)butan-1-one | 20200 | FALSE |
| 3-Methyl-1-adamantanecarboxylic acid | 20200 | FALSE |
| 3-Methyl-2-oxovaleric acid | 20200 | FALSE |
| 3-Methylhistamine | 20101 | TRUE |
| 3-Methylindole | 20101 | TRUE |
| 3-Methylquinolin-4-amine | 10111 | TRUE |
| 3-Methylxanthine | 20200 | TRUE |
| 3-Nitrophenylhydrazine | 20200 | FALSE |
| 3-Oxocholic acid | 20101 | TRUE |
| 3-Phenoxypropionic acid | 20201 | TRUE |
| 3-Piperidin-4-ylpropanoic acid | 20211 | TRUE |
| 3-Tert-Butyl-4-hydroxyanisole | 20222 | TRUE |
| 3-β-hydroxy-20-oxopregn-5-en-17-α-yl sulfate | 20120 | TRUE |
| 4-(2,5-Dimethylphenyl)-4-oxobutanoic acid | 20222 | TRUE |
| 4-(3,4-Dimethylphenoxy)butanoic acid | 10210 | TRUE |
| 4-(3-Methyl-5-oxo-4,5-dihydro-1H-pyrazol-1-yl)benzenesulfonic acid | 20110 | TRUE |
| 4-(Butylamino)benzoic acid | 20211 | TRUE |
| 4-(Dimethylamino)-N-(1,3-thiazol-2-yl)benzamide | 20102 | FALSE |
| 4-(Hydroxymethyl)benzenesulfonic acid | 10210 | TRUE |
| 4,4'-(Oxydiethylene)bis(morpholine) | 20221 | TRUE |
| 4,4,6-Trimethyl-1-(4-nitrophenyl)-3,4-dihydro-2(1H)-pyrimidinethione | 20102 | FALSE |
| 4,4'-Propane-2,2-diylbis(2,6-dimethylphenol) | 10201 | FALSE |
| 4-[(Isobutylamino)methyl]benzoic acid | 20002 | FALSE |
| 4-[[(2S)-4,4-difluoro-2-(pyrrolidin-1-ylmethyl)pyrrolidin-1-yl]methyl]-5-(2-methoxyphenyl)-1H-pyrazole | 20200 | FALSE |
| 4-Acetyl-N-[3-(2-amino-2-oxoethoxy)phenyl]-3-ethyl-5-methyl-1H-pyrrole-2-carboxamide | 20002 | FALSE |
| 4-Amino-2-methyl-5-pyrimidinemethanol | 20211 | TRUE |
| 4-Aminoantipyrine | 20222 | TRUE |
| 4-Aminohippuric acid | 20220 | TRUE |
| 4-Aminomethyltetrahydropyran | 20002 | FALSE |
| 4-Aminophenol | 20200 | TRUE |
| 4-Butylmorpholine | 10210 | TRUE |
| 4-formyl Indole | 20210 | TRUE |
| 4-Heptanone | 20200 | TRUE |
| 4-Hydroxy-3-(3-methylbut-2-enyl)benzoic acid | 10222 | TRUE |
| 4-Hydroxybenzaldehyde | 20020 | TRUE |
| 4-Hydroxy-N-methylbenzamide | 20220 | TRUE |
| 4-Isopropyl-3-methylphenol | 10222 | TRUE |
| 4-Ketoretinal | 20210 | TRUE |
| 4-Methoxycinnamic acid | 20020 | TRUE |
| 4-Methyl-1-phenylpentan-3-one | 10210 | TRUE |
| 4-Methyl-N-(4-nitrophenyl)-1-piperazinecarbothioamide | 20101 | FALSE |
| 4-phenyl-1,5-diazecan-2-one | 20200 | FALSE |
| 4-Pregnen-17.alpha., 20.beta.-diol-3-one-20-sulfate | 20120 | TRUE |
| 4-Pregnen-6.beta.,11.beta.,17,21-tetrol-3,20-dione | 10112 | TRUE |
| 4-Prop-1-enylveratrole | 20011 | TRUE |
| 4-Pyridinecarboximidamide | 20200 | FALSE |
| 4-Pyridoxic acid | 20201 | TRUE |
| 5-(2-Oxohexahydro-1H-thieno[3,4-D]imidazol-4-yl)pentanohydrazide | 10102 | FALSE |
| 5-(3-fluorophenyl)-3-[[3-(4-propan-2-yloxyphenyl)-1,2,4-oxadiazol-5-yl]methyl]-3a,6a-dihydropyrrolo[3,4-d]triazole-4,6-dione | 20200 | FALSE |
| 5-(Difluoromethoxy)-2-[[(3,4-dimethoxy-2-pyridinyl)methyl]thio]-1H-benzimidazole | 10221 | TRUE |
| 5(S),14(R)-Lipoxin B4 | 20120 | TRUE |
| 5(S),6(R)-Lipoxin A4 | 20110 | TRUE |
| 5,6-Dihydroxy-8Z,11Z,14Z-eicosatrienoic acid | 20220 | TRUE |
| 5.alpha.-Androstan-3.beta.-ol-17-one sulfate | 20220 | TRUE |
| 5.alpha.-Pregnan-3.alpha.,17-diol-20-one 3-sulfate | 20120 | TRUE |
| 5-[(6,7,8-trimethoxyquinazolin-4-yl)amino]pentan-1-ol | 20200 | FALSE |
| 5-Benzofurancarboxylic acid, 2,3-dihydro-2-(1-hydroxy-1,5-dimethyl-4-hexen-1-yl)-7-(3-methyl-2-buten-1-yl)- | 20102 | FALSE |
| 5-Bromo-2-[(cyclopropylcarbonyl)amino]benzoic acid | 20120 | TRUE |
| 5-Dodecenoic acid | 20202 | TRUE |
| 5-ethyl-1-(3-ethylphenyl)triazole-4-carboxylic acid | 20200 | FALSE |
| 5-Hydroxyindoleacetic acid | 20220 | TRUE |
| 5-Hydroxytryptophol | 20202 | TRUE |
| 5-Isoprostaglandin-F2.alpha.-VI | 20110 | TRUE |
| 5-Isoquinolinol | 20210 | TRUE |
| 5-Methoxytryptophan | 20102 | TRUE |
| 5-Methyl-2-phenyl-1H-imidazole-4-carboxylic acid | 10012 | TRUE |
| 5-Methylcytosine | 20212 | TRUE |
| 5'-Methylthioadenosine | 20201 | TRUE |
| 5-Oxo-6E,8Z,11Z,14Z-eicosatetraenoic acid | 10210 | TRUE |
| 5S-Hydroxy-6E,8Z,11Z-eicosatrienoic acid | 20220 | TRUE |
| 5-Tetradecynoic acid | 20211 | TRUE |
| 6-(4-Methoxyphenyl)-2-sulfanylidene-2,3-dihydropyrimidin-4(1H)-one | 20200 | FALSE |
| 6-(Methylamino) purine | 20202 | TRUE |
| 6.beta.-Hydroxydexamethasone | 10112 | TRUE |
| 6-[4-(1,3-benzodioxole-5-carbonyl)piperazin-1-yl]-2-(4-propan-2-ylphenyl)pyridazin-3-one | 20120 | TRUE |
| 6-Amino-2-benzoxazolinone | 20201 | FALSE |
| 6-Benzylaminouracil | 20200 | FALSE |
| 6-Heptynoic acid | 10012 | TRUE |
| 6-Hydroxy-3,4-dihydro-1(2H)-naphthalenone | 20020 | TRUE |
| 6-Methoxychromanone | 20002 | FALSE |
| 6-Methylquinoline | 10210 | TRUE |
| 6-Trans-12-epi-Leukotriene B4 | 20220 | TRUE |
| 7-(1H-Imidazol-1-yl)-5,6-dihydro-2-naphthalenecarboxylic acid | 10210 | TRUE |
| 7-(4-fluoroanilino)-2-(2-morpholin-4-yl-2-oxoethyl)-[1,2,4]triazolo[4,3-a]pyrimidin-3-one | 20200 | FALSE |
| 7-(furan-2-carbonyl)-2-(2-methoxyethyl)-5,6,8,9-tetrahydro-[1,2,4]triazolo[4,3-d][1,4]diazepin-3-one | 20200 | FALSE |
| 7,7-Dimethyl-(5Z,8Z)-eicosadienoic acid | 20200 | FALSE |
| 7.alpha.,24(S)-Dihydroxy-4-cholesten-3-one | 20110 | TRUE |
| 7.alpha.-Hydroxy-3-oxo-4-cholestenoic acid | 20210 | TRUE |
| 7-[[5-(4-methylphenyl)-1H-pyrazol-4-yl]methyl]-7-azaspiro[3.5]nonan-3-ol | 20200 | FALSE |
| 7a-Hydroxy-3-oxo-5b-cholanoic acid | 20101 | TRUE |
| 7-Hydroxy-8,11,13-abietatrien-19-oic acid | 20101 | FALSE |
| 7-Hydroxykaurenolide | 20211 | TRUE |
| 7-Hydroxymethotrexate | 20102 | FALSE |
| 7-Ketocholesterol | 20101 | TRUE |
| 7-Methylxanthine | 20101 | TRUE |
| 7-Oxopimara-8(14),15-dien-20-oic acid | 10112 | TRUE |
| 8,11,14-Eicosatrienoic acid | 20201 | TRUE |
| 8,11-eicosadiynoic acid | 20221 | TRUE |
| 8-Chlorotheophylline | 20220 | TRUE |
| 8S,15S-Dihydroxy-5Z,9E,11Z,13E-eicosatetraenoic acid | 20210 | TRUE |
| 9(11)-Dehydromanogenin | 10102 | FALSE |
| 9-(5-O-Methylpentofuranosyl)-1,9-dihydro-6H-purin-6-one | 20220 | TRUE |
| 9,12-Octadecadiynoic Acid | 20212 | TRUE |
| 9-Hydroxy-10E,12Z-octadecadienoic acid | 20110 | TRUE |
| 9-Hydroxynonanoic acid | 20220 | TRUE |
| 9-Hydroxyrisperidone | 10121 | TRUE |
| 9-OAHSA | 10120 | TRUE |
| 9-Oxo-11-(3-pentyl-2-oxiranyl)-10E-undecenoic acid | 20210 | TRUE |
| 9-OxoODE | 20110 | TRUE |
| 9-OxoOTrE | 20110 | TRUE |
| 9-Oxoprosta-10,12Z,14E-trienoic acid | 20210 | TRUE |
| 9-Phenanthrol | 20200 | FALSE |
| 9Z,11E,13E-Octadecatrienoic acid | 20220 | TRUE |
| AC1L6MM4 | 20200 | FALSE |
| AC1L7UIK | 10212 | TRUE |
| Acesulfame | 20200 | TRUE |
| Acetaminophen | 20201 | TRUE |
| Acetaminophen glucuronide | 20101 | FALSE |
| Acetoin | 20200 | TRUE |
| Acetylsulfamethoxazole | 20220 | TRUE |
| Adenine | 20002 | TRUE |
| Adenosine | 20101 | TRUE |
| Adenylsuccinic acid | 20200 | FALSE |
| Ala Glu Ile Lys | 10102 | FALSE |
| Aldosterone | 20102 | TRUE |
| Allocholic acid | 20201 | TRUE |
| Allocystathionine | 20200 | TRUE |
| All-trans-retinoic acid | 20101 | TRUE |
| alpha-curcumene | 20112 | TRUE |
| Alpha-ketoisovaleric acid | 20200 | FALSE |
| Alpha-N-phenylacetyl-L-glutamine | 20201 | TRUE |
| Aminocaproic acid | 20202 | TRUE |
| Amoxicillin | 20201 | FALSE |
| Ampicillin | 20121 | TRUE |
| Amprenavir | 20102 | FALSE |
| Androstan-3-ol-17-one 3-glucuronide | 20221 | TRUE |
| Androstenedione | 20102 | TRUE |
| Androsterone | 20201 | FALSE |
| Androsterone glucuronide | 20220 | TRUE |
| Angiotensin I-Converting Enzyme Substrate | 20102 | FALSE |
| Aniline | 20200 | TRUE |
| Antibiotic OM 173.alpha.2 | 20200 | FALSE |
| Antipyrine | 10212 | TRUE |
| Arachidonic sulfonic acid | 20200 | FALSE |
| Arg Leu Asn Arg | 20102 | FALSE |
| Arg Thr Ala Arg | 20200 | FALSE |
| Arsenobetaine | 20021 | TRUE |
| Asn Asp Gly Val Glu | 20102 | FALSE |
| Aspartame | 20201 | TRUE |
| Asymmetric dimethylarginine | 20201 | TRUE |
| Atenolol | 20101 | TRUE |
| Atorvastatin | 20122 | TRUE |
| Azacyclotridecan-2-one | 10222 | TRUE |
| Azelaic acid | 20201 | FALSE |
| Benzamide | 20002 | TRUE |
| Benzenesulfonic acid | 20020 | TRUE |
| Benzimidazole | 10211 | TRUE |
| Benzocaine | 20201 | TRUE |
| Benzoic acid | 20201 | TRUE |
| Benzotriazole | 10221 | TRUE |
| Benzyl alcohol | 20201 | TRUE |
| Betaine | 20201 | TRUE |
| Beta-Leucine | 20102 | FALSE |
| Betamethasone | 20110 | TRUE |
| Betamethasone 9,11-epoxide | 10212 | TRUE |
| Beta-N-acetylglucosamine | 20200 | FALSE |
| Bilirubin | 10210 | TRUE |
| Biliverdin | 10120 | TRUE |
| Bis(2-ethylhexyl) adipate | 20220 | TRUE |
| Bis(3,5,5-trimethylhexyl) phthalate | 20121 | TRUE |
| Bis(p-methylbenzylidene)sorbitol | 20121 | TRUE |
| Boldenone sulfate | 20110 | TRUE |
| Butanoic acid, 3-methyl-, 2-hydroxy-1-[hydroxy(7-methoxy-2-oxo-2H-1-benzopyran-6-yl)methyl]-2-methylpropyl ester | 20101 | FALSE |
| Butyryl-L-carnitine | 10222 | TRUE |
| Caffeine | 20202 | TRUE |
| Canthaxanthin | 10220 | TRUE |
| Capric acid | 20201 | TRUE |
| Carboxyibuprofen | 10220 | TRUE |
| Carnosic acid | 20101 | FALSE |
| CATECHIN TETRAMETHYLETHER | 20002 | FALSE |
| CE 18:2 | 20112 | TRUE |
| CE 20:4 | 20111 | TRUE |
| Cefdinir | 20200 | FALSE |
| Cefotaxime | 20220 | TRUE |
| Cefuroxime | 20220 | TRUE |
| Cephalexin | 20200 | TRUE |
| Cer 18:1;O2/16:0 | 20211 | TRUE |
| Cer 18:2;O2/16:0 | 20111 | TRUE |
| Cer 28:7;O2 | 20200 | TRUE |
| Cer 28:8;O3 | 20200 | TRUE |
| Cer 32:1;O2 | 20201 | FALSE |
| Cer 34:6;O3 | 20200 | TRUE |
| Cer 36:2;O2 | 20201 | FALSE |
| Cer 40:2;O2 | 20102 | FALSE |
| Cer 42:1;O2 | 20202 | FALSE |
| Cer 42:2;O2 | 20202 | TRUE |
| Cer 42:3;O2 | 20102 | TRUE |
| CerP 28:0;O2 | 20101 | TRUE |
| CerP 28:1;O2 | 20102 | FALSE |
| CerP 28:2;O2 | 10202 | TRUE |
| CerP 30:0;O2 | 20202 | TRUE |
| CerP 30:1;O2 | 20202 | TRUE |
| CerP 32:0;O2 | 20202 | TRUE |
| CerP 32:1;O2 | 20202 | TRUE |
| CerP 32:2;O2 | 10201 | TRUE |
| CerP 34:0;O2 | 10202 | TRUE |
| CerP 34:1;O2 | 20101 | TRUE |
| Cetirizine | 10221 | TRUE |
| CETRIMONIUM | 20210 | TRUE |
| Cetylpyridinium | 10220 | TRUE |
| CHEBI:69439 | 10220 | TRUE |
| Cholesta-4,6-dien-3-one | 20200 | FALSE |
| Cholestenone | 20101 | TRUE |
| Cholesterol sulfate | 20220 | TRUE |
| Cholic acid | 20202 | TRUE |
| Choline | 20220 | TRUE |
| Cinnamaldehyde | 20200 | FALSE |
| Ciprostene | 20210 | TRUE |
| cis-4,10,13,16-Docosatetraenoic Acid | 20120 | TRUE |
| Cis-4,7,10,13,16,19-Docosahexaenoic acid | 20220 | TRUE |
| Cis-5,8,11,14,17-Eicosapentaenoic acid | 20210 | TRUE |
| Clopidogrel carboxylic acid | 10111 | TRUE |
| CMPF | 20220 | TRUE |
| Coniine | 20012 | TRUE |
| Cortexolone | 20101 | TRUE |
| Corticosterone | 20202 | TRUE |
| Cortisol | 20202 | TRUE |
| Cortisol 21-sulfate | 20121 | TRUE |
| Cotinine | 20201 | TRUE |
| Creatinine | 20202 | TRUE |
| Cuminaldehyde | 10210 | TRUE |
| Cyclo(isoleucylprolyl) | 10220 | TRUE |
| Cyclo(leucylprolyl) | 10221 | TRUE |
| Cyclohexylsulfamate | 20120 | TRUE |
| Cymoxanil | 20220 | TRUE |
| Cys-Trp | 20101 | FALSE |
| Cytidine | 20200 | TRUE |
| Cytosine | 20200 | FALSE |
| Deoxycholic acid glycine conjugate | 20200 | TRUE |
| Deoxyguanosine | 20201 | TRUE |
| Desaminosulfamethazine | 20200 | FALSE |
| Desmethyldoxepin | 10220 | TRUE |
| Dexamethasone | 10211 | TRUE |
| Dexpanthenol | 10221 | TRUE |
| DG 34:3 | 20102 | FALSE |
| DG 34:4 | 20102 | FALSE |
| DG 36:3 | 20101 | TRUE |
| DG 36:4 | 20101 | TRUE |
| DG 36:5 | 20101 | FALSE |
| DG 38:6 | 20102 | TRUE |
| DG 42:7 | 20101 | TRUE |
| DG 44:8 | 20102 | TRUE |
| DG O-28:2 | 20200 | FALSE |
| DG O-28:5 | 10202 | FALSE |
| DG O-30:2 | 20101 | TRUE |
| DG O-30:4 | 10202 | TRUE |
| DG O-30:5 | 20202 | FALSE |
| DG O-32:3 | 20202 | TRUE |
| DG O-40:8 | 10201 | TRUE |
| DG O-42:7 | 20200 | TRUE |
| D-Galactose | 20200 | FALSE |
| D-Glucose | 20200 | TRUE |
| D-Glucurono-6,3-lactone | 20200 | TRUE |
| D-Glutamine | 20200 | TRUE |
| DGTS 36:1 | 20200 | TRUE |
| Diatrizoic acid | 10220 | TRUE |
| Diazepam | 20111 | TRUE |
| Dibenzepin | 20101 | FALSE |
| Diclofenac (sodium salt) | 20220 | TRUE |
| Didecyl hydrogen phosphate | 20120 | TRUE |
| Diethanolamine | 20101 | TRUE |
| Diethyltoluamide | 20020 | TRUE |
| Dihydromorphine | 20212 | TRUE |
| Diisopropanolamine | 20222 | TRUE |
| Dimethyl sulfone | 20201 | TRUE |
| Diosgenin | 20102 | FALSE |
| Diphenyl phenylphosphonate | 10120 | TRUE |
| Diphenyl phosphate | 20220 | TRUE |
| Diphenylguanidine | 10220 | TRUE |
| DL-2-aminooctanoic acid | 20201 | TRUE |
| DL-2-hydroxy stearic acid | 20212 | TRUE |
| Docosahexaenoic Acid ethyl ester | 10210 | TRUE |
| Docosanamide | 10211 | TRUE |
| Dodecanedioic acid | 20201 | TRUE |
| Dodecanoic acid | 20201 | TRUE |
| Dodecylbenzenesulfonic acid | 20222 | TRUE |
| Dodemorph | 20212 | TRUE |
| Dolastatin 10 | 20202 | FALSE |
| Doxepin | 10220 | TRUE |
| Doxylamine | 20102 | TRUE |
| D-Phenyllactic acid | 20220 | TRUE |
| D-Tagatose | 10201 | TRUE |
| Ectoine | 20200 | FALSE |
| Enalapril | 20212 | TRUE |
| Epi-inositol | 10202 | TRUE |
| Erucamide | 20210 | TRUE |
| ERYTHROMYCIN | 10220 | TRUE |
| Estriol | 20200 | FALSE |
| estrone 3-sulfate | 20110 | TRUE |
| Eszopiclone N-oxide | 20112 | TRUE |
| Ethosuximide | 20200 | FALSE |
| Ethoxysulfuron | 20102 | FALSE |
| ethyl 2-[3-(4-methylpiperidin-1-yl)sulfonyl-2-oxopyridin-1-yl]acetate | 10210 | TRUE |
| ethyl 4-[(Z)-(6-hydroxy-7-methyl-3-oxo-1-benzofuran-2-ylidene)methyl]piperazine-1-carboxylate | 20200 | FALSE |
| Ethyl 4-amino-2-(methylthio)-1,3-thiazole-5-carboxylate | 20200 | FALSE |
| Etofylline | 10220 | TRUE |
| Farnesyl acetone | 20110 | TRUE |
| Fenamidone | 20112 | TRUE |
| Fenuron | 10221 | TRUE |
| Fingolimod | 20002 | FALSE |
| Fluconazole | 20221 | TRUE |
| Foetidin | 20111 | TRUE |
| Fructose | 20220 | TRUE |
| furan-2-yl-[3-(2H-tetrazol-5-yl)piperidin-1-yl]methanone | 20200 | FALSE |
| Gabapentin related bis-nitrile | 20200 | FALSE |
| Galactitol | 20221 | TRUE |
| Gamma-aminobutyric acid | 20002 | FALSE |
| gamma-Linolenoyl dopamine | 20200 | FALSE |
| Gemcitabine hydrochloride | 20200 | FALSE |
| Givinostat | 20101 | FALSE |
| Gln-Cys | 10112 | TRUE |
| Gluconic acid | 10202 | TRUE |
| Glucosamine | 10102 | TRUE |
| Glu-Gly-Arg | 20101 | FALSE |
| Glutamine phenylthiohydantoin | 20101 | FALSE |
| Glycerol tricaprylate | 10112 | TRUE |
| Glycocholic acid | 20200 | TRUE |
| Glycyl-L-leucine | 20200 | TRUE |
| Gly-His | 20101 | FALSE |
| Guaiacol | 20201 | TRUE |
| Guanosine | 20220 | TRUE |
| Heptadecanoic acid | 20102 | TRUE |
| hexadeca-9-en-1-ol | 20200 | FALSE |
| Hexadecanedioic acid | 20202 | TRUE |
| Hexaethylene glycol | 20200 | FALSE |
| Hexanoyl-L-carnitine | 10222 | TRUE |
| HexCer 18:1;O2/16:0 | 20211 | TRUE |
| HexCer 30:2;O2 | 20002 | FALSE |
| HexCer 32:4;O3 | 10102 | TRUE |
| HexCer 42:3;O2 | 20102 | TRUE |
| HexCer 42:5;O3 | 20101 | TRUE |
| Hexylparaben | 20200 | FALSE |
| Hippuric acid | 20220 | TRUE |
| Homoveratric acid | 20200 | TRUE |
| Hydrocortisone 21-hemisuccinate | 10102 | FALSE |
| Hydromorphone | 10221 | TRUE |
| Hydroxydehydronifedipinecarboxylic acid | 10221 | TRUE |
| Hydroxymetronidazole | 20221 | TRUE |
| Hydroxyoctanoic acid | 20201 | TRUE |
| Hydroxypropionic acid | 20200 | FALSE |
| Hypaphorine | 20212 | TRUE |
| Hypoxanthine | 20220 | TRUE |
| Ibuprofen .beta.-D-glucuronide | 20212 | TRUE |
| Ile Phe Gln Glu | 20102 | FALSE |
| Ile-Leu | 10222 | TRUE |
| Ile-Pro | 10221 | TRUE |
| Indole | 20201 | TRUE |
| Indole-3-carbinol | 20200 | TRUE |
| Indole-7-carboxaldehyde | 10220 | TRUE |
| Indoleacetaldehyde | 20021 | TRUE |
| Indoleacrylic acid | 20202 | TRUE |
| Indolelactic acid | 20210 | TRUE |
| Indoline | 10212 | TRUE |
| Indoxyl sulfate | 20220 | TRUE |
| Inosine | 20200 | TRUE |
| Ipecac (Cephaeline) | 20102 | FALSE |
| Isoguanine | 20002 | TRUE |
| Isopentenyl adenosine | 10221 | TRUE |
| Isoquinoline | 10210 | TRUE |
| Isovalerylglycine | 20201 | TRUE |
| Kaempferol | 10102 | TRUE |
| KHIVORIN | 10102 | FALSE |
| Kynurenic acid | 20202 | TRUE |
| L(-)-Nicotine pestanal | 20201 | TRUE |
| L,L-Cyclo(leucylprolyl) | 20222 | TRUE |
| L-Acetylcarnitine | 20201 | TRUE |
| L-a-Lysophosphatidylserine | 20102 | FALSE |
| L-Arachidonoylcarnitine | 20120 | TRUE |
| LARIXOL ACETATE | 20212 | TRUE |
| L-Aspartyl-L-phenylalanine | 20201 | TRUE |
| Latanoprost ethylamide | 20200 | FALSE |
| Laurylsulfuric acid | 20221 | TRUE |
| L-Carnitine | 20201 | TRUE |
| Leu Ile Asp Arg | 10111 | TRUE |
| Leu Leu | 10220 | TRUE |
| Leu Leu Val Val Ala | 10102 | FALSE |
| Leucinic acid | 20221 | TRUE |
| Leu-Phe | 20221 | TRUE |
| Levofloxacin | 20201 | TRUE |
| Levoglucosan | 20210 | TRUE |
| Levulinic acid | 20221 | TRUE |
| L-Histidine | 20200 | TRUE |
| Lidocaine | 10221 | TRUE |
| Linoleic acid | 20202 | TRUE |
| Lithocholylglycine | 20120 | TRUE |
| L-Kynurenine | 20201 | TRUE |
| L-Leucine | 20211 | TRUE |
| L-Norleucine | 20200 | FALSE |
| LPA 18:1 | 20210 | TRUE |
| LPA 20:4 | 20220 | TRUE |
| LPC 14:0 | 20212 | TRUE |
| LPC 14:1 | 20202 | TRUE |
| LPC 14:2 | 20202 | TRUE |
| LPC 14:3 | 20200 | TRUE |
| LPC 16:0 | 20212 | TRUE |
| LPC 16:1 | 10201 | TRUE |
| LPC 18:0 | 20202 | TRUE |
| LPC 18:1 | 10201 | TRUE |
| LPC 18:2 | 20201 | TRUE |
| LPC 18:3 | 20201 | TRUE |
| LPC 20:0 | 20202 | TRUE |
| LPC 20:1 | 20201 | TRUE |
| LPC 20:2 | 20202 | TRUE |
| LPC 20:3 | 20200 | TRUE |
| LPC 20:4 | 20102 | TRUE |
| LPC 22:0 | 20202 | TRUE |
| LPC 22:1 | 20202 | TRUE |
| LPC 22:2 | 10202 | TRUE |
| LPC 22:3 | 10202 | FALSE |
| LPC 22:4 | 20201 | TRUE |
| LPE 16:0 | 20222 | TRUE |
| LPE 16:1 | 20211 | TRUE |
| LPE 18:0 | 10202 | TRUE |
| LPE 18:1 | 20212 | TRUE |
| LPE 18:2 | 20211 | TRUE |
| LPE 18:3 | 20221 | TRUE |
| LPE 20:0 | 20200 | TRUE |
| LPE 20:1 | 20201 | TRUE |
| LPE 20:2 | 20202 | TRUE |
| LPE 20:3 | 20211 | TRUE |
| LPE 20:4 | 20212 | TRUE |
| LPE 22:0 | 20200 | TRUE |
| LPE 22:1 | 20200 | TRUE |
| LPE 22:4 | 20202 | TRUE |
| LPE O-14:0 | 20201 | TRUE |
| LPE O-16:0 | 20101 | TRUE |
| LPE O-16:1 | 20210 | TRUE |
| LPE O-16:2 | 20200 | TRUE |
| LPE O-18:1 | 20220 | TRUE |
| LPE O-18:2 | 20220 | TRUE |
| LPE O-18:3 | 10102 | TRUE |
| LPE O-20:1 | 20201 | TRUE |
| LPE O-20:2 | 20101 | FALSE |
| LPG 16:0 | 20200 | FALSE |
| LPG 18:1 | 20210 | TRUE |
| LPG 20:4 | 20110 | TRUE |
| L-Phenylalanine | 20220 | TRUE |
| L-phenylalanyl-L-proline | 20220 | TRUE |
| LPI 18:0 | 20200 | TRUE |
| LPI 18:1 | 20110 | TRUE |
| LPI 20:4 | 20210 | TRUE |
| L-Pipecolic acid | 20200 | TRUE |
| LPS 18:0 | 20120 | TRUE |
| LPS 18:1 | 20111 | TRUE |
| LPS 20:0 | 20200 | TRUE |
| L-Tryptophan | 20221 | TRUE |
| L-Tyrosine | 20200 | TRUE |
| Lys Gly Ala Glu Lys | 20201 | FALSE |
| Lys Ile Gln Asp Lys | 10102 | FALSE |
| Lys Val Ile Arg | 20101 | FALSE |
| MCI-186 | 10220 | TRUE |
| m-Coumaric acid | 20200 | TRUE |
| m-Cresol | 20201 | TRUE |
| Medroxyprogesterone | 20101 | TRUE |
| Mepivacaine | 20222 | TRUE |
| Meropenem | 10221 | TRUE |
| Mesoridazine | 10111 | TRUE |
| Methyl 3-(3,3-difluorocyclobutyl)-3-oxopropanoate | 20002 | FALSE |
| Methyl 4-[(6-deoxy-?-L-mannopyranosyl)oxy]-3,5-dimethoxybenzoate | 20200 | FALSE |
| Methyl 6-(acetyloxy)-1,7,11-trihydroxyabieta-8,11,13-trien-18-oate | 20102 | FALSE |
| Methyl morpholine-3-carboxylate | 20211 | TRUE |
| methyl N-[4-[2-(furan-2-ylmethylamino)-2-oxoethyl]-1,3-thiazol-2-yl]carbamate | 20200 | FALSE |
| Metochlopramide | 10222 | TRUE |
| Metoprolol | 20202 | TRUE |
| Metoprolol acid | 10211 | TRUE |
| Metronidazole | 20221 | TRUE |
| Mevalonic acid | 20200 | FALSE |
| MG 14:2 | 20200 | TRUE |
| MG 14:3 | 20200 | TRUE |
| MG 14:4 | 20200 | TRUE |
| MG 16:2 | 20200 | TRUE |
| MG 16:3 | 20200 | TRUE |
| MG 16:4 | 20200 | TRUE |
| MG 18:2 | 20200 | TRUE |
| MG 18:3 | 20200 | TRUE |
| MG 18:4 | 20200 | TRUE |
| MG 20:2 | 20200 | TRUE |
| MG 20:3 | 20200 | TRUE |
| MG 20:4 | 20200 | TRUE |
| MG 22:3 | 20200 | TRUE |
| MG 22:4 | 20200 | TRUE |
| Midazolam | 20211 | TRUE |
| Mitoxantrone | 10102 | FALSE |
| Mono-2-ethylhexyl phthalate | 20112 | TRUE |
| Monobutyl phthalate | 20212 | TRUE |
| Monoethylglycylxylidide (MEGX) | 10222 | TRUE |
| Monomethyl glutaric acid | 20200 | FALSE |
| Myristic acid | 20202 | TRUE |
| N-(1,3,6-trimethyl-2-oxobenzimidazol-5-yl)furan-2-carboxamide | 20200 | FALSE |
| N-(1,5-Dimethyl-3-oxo-2-phenyl-2,3-dihydro-1H-pyrazol-4-yl)propanamide | 20222 | TRUE |
| N-(14-Methylpentadecanoyl)phenylalanine | 20112 | TRUE |
| N-(2,6-Dimethylphenyl)-1-methyl-2-piperidinecarboxamide | 10221 | TRUE |
| N-(2,6-Dimethylphenyl)-1-piperazineacetamide | 10221 | TRUE |
| N-(3-methylphenyl)-2-(4-morpholin-4-yl-1-oxophthalazin-2-yl)acetamide | 20110 | TRUE |
| N-(4-Acetylphenyl)-N'-(4-pyridinyl)urea | 20200 | FALSE |
| N-(4-ethoxyphenyl)-1-methylpiperidine-3-carboxamide | 20210 | TRUE |
| N-(4-methoxy-2-methylphenyl)-2-pyrrolidin-1-yl-5,6,7,8-tetrahydroquinazoline-6-carboxamide | 20200 | FALSE |
| N-(4-methoxyphenyl)-2-methyl-7-oxoazepane-2-carboxamide | 20210 | TRUE |
| N-(4-Methoxyphenyl)-2-oxo-2H-chromene-3-carboxamide | 10122 | TRUE |
| N'-(4-Methylbenzenesulfonyl)benzohydrazide | 10102 | FALSE |
| N-(Tert-Butyl)-2-piperidinecarboxamide | 20220 | TRUE |
| N,1-Diethyl-2-oxo-1,2-dihydrobenzo[cd]indole-6-sulfonamide | 20200 | FALSE |
| N,N'-Dicyclohexylurea | 10222 | TRUE |
| N,N-Dimethylaniline | 20201 | TRUE |
| N,N-Dimethylguanosine | 10222 | TRUE |
| N.alpha.-Benzoyl-DL-arginine-4-nitroanilide | 20201 | FALSE |
| N-[(2,4-difluorophenyl)methyl]-2-(1-ethylpyrrolo[2,3-b]pyridin-3-yl)-1,3-thiazole-4-carboxamide | 20200 | FALSE |
| N-[(5-propan-2-yl-1,2,4-oxadiazol-3-yl)methyl]benzamide | 20210 | TRUE |
| N-[2-(6-oxopyridazin-1-yl)ethyl]-4-(trifluoromethoxy)benzenesulfonamide | 20200 | FALSE |
| N-[2-(diethylamino)ethyl]-5-methyl-4-(4-methylpiperazin-1-yl)thieno[2,3-d]pyrimidine-6-carboxamide | 20200 | FALSE |
| N-[3-(diethylamino)propyl]-2-[(4-methylphenyl)methyl]-3-oxo-1H-isoindole-1-carboxamide | 20200 | FALSE |
| N-[3-(methoxymethyl)-6,7,8,9-tetrahydro-5H-[1,2,4]triazolo[4,3-a]azepin-7-yl]furan-3-carboxamide | 20200 | FALSE |
| N-[3,5-bis(trifluoromethyl)phenyl]-6-oxo-1H-pyridazine-3-carboxamide | 20200 | FALSE |
| N2-(1-Oxo-4-phenylbutyl)-L-glutamine | 20200 | FALSE |
| N-Acetyl-D-galactosamine 4-sulfate | 20222 | TRUE |
| N-Acetyl-DL-tryptophan | 20211 | TRUE |
| N-Acetyl-DL-valine | 20222 | TRUE |
| N-Acetyl-D-tryptophan | 20200 | FALSE |
| N-Acetyl-L-phenylalanine | 20111 | TRUE |
| N-Acetylputrescine | 20101 | TRUE |
| N-Acetylserotonin | 20002 | TRUE |
| N-Acetylsulfamethoxazole | 10222 | TRUE |
| N-ACETYLTRYPTAMINE | 20210 | TRUE |
| N-Acetyltryptophan | 20220 | TRUE |
| Naringenin | 10102 | TRUE |
| N'-Benzoyl-4-nitrobenzohydrazide | 20200 | FALSE |
| N-benzyl-5-methyl-4-oxopyrazolo[1,5-a]quinoxaline-7-carboxamide | 20200 | FALSE |
| N-cis-tetradec-9Z-enoyl-L-Homoserine lactone | 20200 | FALSE |
| N-cyclopentyl-1-(5-pyrimidin-5-yl-[1,3]thiazolo[5,4-b]pyridin-2-yl)piperidine-4-carboxamide | 20200 | FALSE |
| N-cyclopropyl-1-oxo-3,4-dihydro-2H-pyrrolo[1,2-a]pyrazine-3-carboxamide | 20200 | FALSE |
| N-Desbutylbupivacaine | 10222 | TRUE |
| Nefiracetam | 10211 | TRUE |
| Nelarabine | 20221 | TRUE |
| Neogrifolin | 20200 | FALSE |
| Neopterin | 20200 | TRUE |
| N-ethyl-1-propanoyl-2,3-dihydroindole-5-carboxamide | 20200 | FALSE |
| N-Ethyl-2-methyl-2-propen-1-amine | 20200 | FALSE |
| N-ethyl-4,6,7-trimethyl-3-oxoquinoxaline-2-carboxamide | 20200 | FALSE |
| N-Ethyl-4-menthane-3-carboxamide | 10222 | TRUE |
| Niacinamide | 20201 | TRUE |
| N-Isobutyl-3-methylbutanamide | 10212 | TRUE |
| N-lactoyl-phenylalanine | 20221 | TRUE |
| N-Methylhydantoin | 20002 | TRUE |
| N-Methylnicotinamide | 20201 | TRUE |
| N-Methylpropionamide | 20200 | FALSE |
| N'-Nitrosoanabasine | 20210 | TRUE |
| N-octadecanoyl-L-Homoserine lactone | 20002 | FALSE |
| n-Octyl caffeate | 20200 | FALSE |
| N-Oleoyl-L-Serine | 20120 | TRUE |
| NONOXYNOL-9 | 10121 | TRUE |
| Nordazepam | 20111 | TRUE |
| Norethindrone | 20211 | TRUE |
| Normetanephrine | 20202 | TRUE |
| Norquetiapine | 10121 | TRUE |
| Norsufentanil | 10211 | TRUE |
| N-Phenyldiethanolamine | 20211 | TRUE |
| N-tert-butyl-4-ethoxypiperidine-1-carboxamide | 20002 | FALSE |
| N-tetradecanoyl-L-Homoserine lactone | 20002 | FALSE |
| Nudifloramide | 20212 | TRUE |
| Nutriacholic acid | 20102 | TRUE |
| o-Cresol | 20220 | TRUE |
| Octadecanedioic acid | 20202 | TRUE |
| Octanoic hydrazide | 20200 | FALSE |
| Octanoylcarnitine | 20221 | TRUE |
| Octodrine | 20211 | TRUE |
| O-Desarylranolazine | 10222 | TRUE |
| O-Desmethylvenlafaxine | 10222 | TRUE |
| Olanzapine | 20110 | TRUE |
| Oleamide | 10210 | TRUE |
| Oleoyl ethylamide | 10120 | TRUE |
| Olivetol | 20200 | FALSE |
| Ortho-hydroxyphenylacetic acid | 20200 | FALSE |
| o-Tyrosine | 20102 | TRUE |
| Oxindole | 20221 | TRUE |
| o-Xylene | 20020 | TRUE |
| Oxypurinol | 20200 | TRUE |
| p-Acetaminobenzaldehyde | 20212 | TRUE |
| Palmitic amide | 10220 | TRUE |
| Palmitoyl-L-carnitine | 20220 | TRUE |
| p-Anisic acid | 20220 | TRUE |
| Panthenol | 20210 | TRUE |
| Pantoprazole | 20222 | TRUE |
| Pantoprazole sulfide | 20222 | TRUE |
| Pantothenic acid | 20201 | TRUE |
| Paracetamol sulfate | 20202 | TRUE |
| PC 28:0 | 20102 | FALSE |
| PC 30:0 | 20212 | TRUE |
| PC 32:1 | 20111 | TRUE |
| PC 32:2 | 20111 | TRUE |
| PC 34:0 | 10102 | FALSE |
| PC 34:1 | 20221 | TRUE |
| PC 34:2 | 20121 | TRUE |
| PC 34:3 | 20121 | TRUE |
| PC 36:3 | 10120 | TRUE |
| PC 36:4 | 20111 | TRUE |
| PC 36:5 | 20111 | TRUE |
| PC 38:3 | 10121 | TRUE |
| PC 38:4 | 20122 | TRUE |
| PC 38:6 | 20122 | TRUE |
| PC 38:7 | 20112 | TRUE |
| PC 40:4 | 20121 | TRUE |
| PC 40:6 | 20112 | TRUE |
| PC O-28:7 | 20200 | TRUE |
| PC O-32:0 | 20202 | FALSE |
| PC O-34:1 | 20202 | FALSE |
| PC O-34:2 | 20102 | FALSE |
| PC O-36:3 | 10102 | FALSE |
| PC O-36:4 | 20112 | TRUE |
| PC O-36:5 | 20111 | TRUE |
| PC O-36:6 | 20101 | FALSE |
| PC O-38:4 | 20111 | TRUE |
| PC O-38:5 | 20112 | TRUE |
| PC O-38:6 | 20102 | TRUE |
| PC O-40:5 | 20101 | TRUE |
| PC O-40:6 | 20111 | TRUE |
| PC(16:0/16:0) | 20201 | TRUE |
| PC(18:1(9Z)/18:1(9Z)) | 20101 | TRUE |
| PE 30:3 | 20200 | TRUE |
| PE 30:7 | 10102 | TRUE |
| PE 34:0 | 20101 | FALSE |
| PE 34:2 | 20101 | TRUE |
| PE 36:1 | 20201 | FALSE |
| PE 36:2 | 20102 | TRUE |
| PE 36:4 | 20101 | TRUE |
| PE 36:5 | 20102 | FALSE |
| PE 36:8 | 20200 | TRUE |
| PE 38:1 | 20101 | FALSE |
| PE 38:2 | 20101 | FALSE |
| PE 38:3 | 20102 | FALSE |
| PE 38:4 | 20201 | TRUE |
| PE 38:5 | 20101 | TRUE |
| PE 38:6 | 20101 | TRUE |
| PE 40:4 | 20101 | TRUE |
| PE 40:6 | 20101 | TRUE |
| PE O-34:3 | 20101 | FALSE |
| PE O-36:5 | 20101 | TRUE |
| PE O-36:6 | 10102 | TRUE |
| PE O-38:5 | 20201 | TRUE |
| PE O-38:6 | 20111 | TRUE |
| PE O-38:7 | 20101 | TRUE |
| PE O-40:7 | 20101 | TRUE |
| PE O-40:8 | 20101 | TRUE |
| PELLETIERINE | 20221 | TRUE |
| Pentaethylene glycol | 20221 | TRUE |
| Perfluorooctanoic acid | 20221 | TRUE |
| PFNA | 20221 | TRUE |
| PG 18:1_18:2 | 20110 | TRUE |
| PGPC | 20221 | TRUE |
| Phe Leu | 20220 | TRUE |
| Phe Val | 10220 | TRUE |
| Phe-Leu | 10222 | TRUE |
| Phenol | 20201 | TRUE |
| Phenoxyacetate | 20220 | TRUE |
| Phenyl dihydrogen phosphate | 20210 | TRUE |
| Phenyl{[4-phenyl-6-(trifluoromethyl)-2-pyrimidinyl]sulfanyl}acetic acid | 20102 | FALSE |
| Phenylacetaldehyde | 20202 | TRUE |
| Phenylalanylphenylalanine | 20221 | TRUE |
| Phenylpropanolamine | 20200 | TRUE |
| Phe-Phe | 10222 | TRUE |
| Phe-Pro | 10121 | TRUE |
| Phe-Trp | 10121 | TRUE |
| Phosphocholine | 20020 | TRUE |
| Phthalic acid | 20200 | FALSE |
| Phthalic anhydride | 20220 | TRUE |
| Phthalide | 10220 | TRUE |
| PI 38:4 | 20212 | TRUE |
| PI 38:5 | 20101 | TRUE |
| Piperacillin | 10220 | TRUE |
| Piperazine-2-carboxylic acid | 20012 | TRUE |
| Pipericine | 20110 | TRUE |
| Piperine | 20220 | TRUE |
| Podocarpic acid | 20200 | FALSE |
| PPA | 20210 | TRUE |
| Prednisolone 21-sulfate | 20122 | TRUE |
| Prednisone | 20112 | TRUE |
| Pregnenolone sulfate | 20120 | TRUE |
| Pro Ile | 10220 | TRUE |
| Pro Tyr Trp | 10120 | TRUE |
| Pro-Leu | 20221 | TRUE |
| Propofol .beta.-D-glucuronide | 20221 | TRUE |
| Propranolol | 20201 | TRUE |
| Prostaglandin H2 | 20110 | TRUE |
| Prostaglandin I2 | 10120 | TRUE |
| Protogenkwanin 4'-glucoside | 10102 | FALSE |
| PS 36:0 | 20200 | FALSE |
| PS 36:1 | 20102 | TRUE |
| PS 36:2 | 20102 | TRUE |
| PS 38:2 | 20102 | TRUE |
| PS 38:3 | 20101 | TRUE |
| PS 40:5 | 20102 | TRUE |
| PS 44:7 | 20200 | TRUE |
| Psychosine | 10120 | TRUE |
| p-Synephrine | 20202 | TRUE |
| p-Tolyl Sulfate | 20220 | TRUE |
| Pyridine | 20200 | TRUE |
| Pyridoxal | 20201 | TRUE |
| Pyridoxamine | 20002 | TRUE |
| PyroGlu-Ile-Arg | 10202 | FALSE |
| Pyroglutamic acid | 20200 | TRUE |
| PyroGlu-Val | 20211 | TRUE |
| Pyrrocaine | 20220 | TRUE |
| Quetiapine | 20221 | TRUE |
| Quetiapine sulfoxide | 10222 | TRUE |
| Quinolin-2-ol | 20221 | TRUE |
| Quinolin-3-ol | 20220 | TRUE |
| Quinolin-8-ol | 10211 | TRUE |
| Rac-N,O-Didesmethylvenlafaxine | 20210 | TRUE |
| Ramipril | 10221 | TRUE |
| Ranolazine | 20221 | TRUE |
| Ranolazine dihydrochloride | 20220 | TRUE |
| Remifentanil | 10211 | TRUE |
| Resminostat (hydrochloride) | 20200 | FALSE |
| Resveratrol-3-O-sulfate | 20110 | TRUE |
| Riboflavin | 20201 | TRUE |
| Ribothymidine | 20210 | TRUE |
| Ricinoleic acid | 20210 | TRUE |
| Risperidone | 10121 | TRUE |
| Rivaroxaban | 20120 | TRUE |
| Rivastigmine | 20211 | TRUE |
| Ropivacaine | 10222 | TRUE |
| Salicylic acid | 20121 | TRUE |
| Salicyluric acid | 20222 | TRUE |
| Sepiapterin | 20201 | TRUE |
| SIB 1757 | 10210 | TRUE |
| SM 18:1;O2/12:0 | 20211 | TRUE |
| SM 18:1;O2/16:0 | 20222 | TRUE |
| SM 18:2;O2/16:0 | 20122 | TRUE |
| SM 18:2;O2/16:1 | 20112 | TRUE |
| SM 28:1;O2 | 20211 | TRUE |
| SM 28:2;O2 | 10212 | TRUE |
| SM 28:8;O2 | 20200 | TRUE |
| SM 30:1;O2 | 20211 | TRUE |
| SM 30:2;O2 | 20121 | TRUE |
| SM 30:8;O3 | 20200 | TRUE |
| SM 32:1;O2 | 20212 | TRUE |
| SM 32:2;O2 | 20122 | TRUE |
| SM 34:1;O2 | 20212 | TRUE |
| SM 34:1;O3 | 20101 | FALSE |
| SM 34:2;O2 | 20122 | TRUE |
| SM 34:2;O3 | 20120 | TRUE |
| SM 34:4;O2 | 20101 | FALSE |
| SM 34:5;O2 | 10102 | FALSE |
| SM 36:2;O2 | 20222 | TRUE |
| SM 36:3;O2 | 20102 | FALSE |
| SM 36:8;O2 | 20200 | TRUE |
| SM 38:1;O2 | 10012 | TRUE |
| SM 38:2;O2 | 20012 | TRUE |
| SM 38:3;O2 | 10102 | FALSE |
| SM 38:4;O2 | 20102 | TRUE |
| SM 40:3;O2 | 20101 | FALSE |
| SM 40:4;O2 | 20002 | FALSE |
| SM 40:7;O2 | 10102 | FALSE |
| SM 40:8;O2 | 20200 | TRUE |
| SM 42:4;O3 | 20111 | TRUE |
| SM 42:5;O2 | 20012 | TRUE |
| SM 42:6;O2 | 10012 | TRUE |
| SM 44:6;O2 | 10111 | TRUE |
| S-Methyl-3-thioacetaminophen | 20220 | TRUE |
| Sorbitol | 20221 | TRUE |
| Sordariol | 20210 | TRUE |
| SPB 16:0;O2 | 20200 | TRUE |
| SPB 16:0;O3 | 20200 | TRUE |
| SPB 18:0;O3 | 20200 | TRUE |
| SPB 18:1;O3 | 20200 | TRUE |
| SPB 18:3;O2 | 20200 | TRUE |
| SPB 18:4;O2 | 20200 | TRUE |
| SPB 20:0;O2 | 20200 | TRUE |
| SPB 20:1;O2 | 20200 | FALSE |
| SPB 20:1;O3 | 20200 | TRUE |
| SPB 20:2;O2 | 20200 | TRUE |
| SPB 22:1;O2 | 20200 | TRUE |
| SPB 22:2;O2 | 20200 | TRUE |
| SPB 22:3;O2 | 20200 | TRUE |
| Spermine | 20201 | TRUE |
| Sphinganine | 20201 | TRUE |
| Sphingosine | 20200 | TRUE |
| SPHINGOSYLPHOSPHORYL CHOLINE | 20120 | TRUE |
| spiro[1,4-dihydroquinoxaline-3,4'-piperidine]-2-one | 20200 | FALSE |
| SQDG 28:6 | 20200 | TRUE |
| Stachydrine | 20220 | TRUE |
| Stearamide | 10210 | TRUE |
| Stearic acid | 20202 | TRUE |
| Stearic Acid ethyl ester | 10102 | FALSE |
| Styrene | 20220 | TRUE |
| Sufentanyl | 20122 | TRUE |
| Sulfamethoxazole | 20220 | TRUE |
| Sulfolithocholic acid | 20102 | TRUE |
| Taurine | 20020 | TRUE |
| Taurocholic acid | 20200 | TRUE |
| Taurodeoxycholic acid | 20200 | TRUE |
| Tazobactam | 10221 | TRUE |
| Terephthalic acid | 20200 | TRUE |
| Tetradecanedioic acid | 20221 | TRUE |
| Tetraethylene glycol | 10220 | TRUE |
| Tetrahydrocortisone | 20201 | TRUE |
| TG 46:1 | 20102 | TRUE |
| TG 46:2 | 20101 | TRUE |
| TG 46:3 | 20101 | TRUE |
| TG 46:5 | 20101 | TRUE |
| TG 48:3 | 20102 | TRUE |
| TG 48:4 | 20101 | TRUE |
| TG 48:6 | 20101 | TRUE |
| Theobromine | 20201 | TRUE |
| Theophylline | 10102 | TRUE |
| Thiamine | 20121 | TRUE |
| Thioridazine | 10122 | TRUE |
| Threonic acid | 20200 | TRUE |
| Thr-Ile-OH | 10102 | FALSE |
| Thr-Leu | 10212 | TRUE |
| Thr-Val-Leu | 20212 | TRUE |
| Thymol | 20202 | TRUE |
| Tolperisone | 20210 | TRUE |
| Torasemide | 10222 | TRUE |
| Torsemide | 20220 | TRUE |
| Tramadol | 20212 | TRUE |
| Tramadol HCl | 20200 | FALSE |
| Trans-3'-Hydroxycotinine | 20220 | TRUE |
| Trans-Cinnamic acid | 20220 | TRUE |
| Tranylcypromine hydrochloride | 10220 | TRUE |
| Triethanolamine | 10220 | TRUE |
| Triethyl citrate | 20200 | FALSE |
| Trigonelline | 20101 | TRUE |
| Trihexyphenidyl | 20221 | TRUE |
| Triisopropanolamine | 10220 | TRUE |
| Trimethoprim | 10221 | TRUE |
| Trimethylamine | 20200 | FALSE |
| Trimethylamine N-oxide | 20200 | FALSE |
| Triphenylphosphine oxide | 10212 | TRUE |
| Tripropylene glycol | 20200 | FALSE |
| Tris(2-ethylhexyl) trimellitate | 20120 | TRUE |
| Tris(butoxyethyl)phosphate | 20220 | TRUE |
| Tropine | 20201 | FALSE |
| Trp Leu | 20220 | TRUE |
| Tryptamine | 20200 | TRUE |
| Tyr Glu Lys Thr Tyr | 10102 | FALSE |
| Tyr-Asn-Lys | 20102 | FALSE |
| Tyr-Leu | 20211 | TRUE |
| Tyr-Phe | 10222 | TRUE |
| U-44069 | 20110 | TRUE |
| Umbelliferone | 20200 | TRUE |
| URAPIDIL | 20221 | TRUE |
| Ureidopropionic acid | 20201 | TRUE |
| Uric acid | 20200 | TRUE |
| Uridine | Uridine | Uridine |
| Urocanic acid | 20021 | TRUE |
| Ursodeoxycholic acid | 20201 | TRUE |
| Vaccenic acid | 20202 | TRUE |
| Venlafaxine | 20221 | TRUE |
| Xanthene-9-carboxylic acid | 20220 | TRUE |
| Xipamide | 10112 | TRUE |
| Z-Ligustilide | 10210 | TRUE |
| α-Hydroxymidazolam | 20221 | TRUE |
| γ-CEHC | 20220 | TRUE |
| Δ2-cis-Hexadecenoic Acid | 20120 | TRUE |
| δ-Valerolactam | 10210 | TRUE |

The five digits give the match with library compounds in the following order: m/z, retention time, mSigma (isotopic pattern), MS/MS spectra and CCS. 2= narrow match; 1 = wide match; 0 = outside of wide match. Thresholds were: m/z (narrow: 2 ppm, wide: 10 ppm); retention time (not used -> always 0); mSigma (narrow: 20, wide: 100, scale 0-∞); MS/MS (narrow: 900, wide: 600, scale 1000-0); CCS (narrow 1.0 %, wide: 5.0%]

**Table S4.1** Ascites Metabolic Signatures in OC II-III, OC IV, and GI Groups through ANOVA

| Metabolite | F.value | P.value | -LOG10(p) | FDR |
| --- | --- | --- | --- | --- |
| 4-Isopropyl-3-methylphenol | 25.877 | 1.37E-05 | 4.8629 | 0.006599 |
| Phenylalanylphenylalanine | 24.465 | 1.90E-05 | 4.7221 | 0.006599 |
| 3-Methoxy-4-(2-methylpropoxy)benzoic acid | 17.739 | 0.000112 | 3.9525 | 0.025879 |
| 2-tert-Butyl-4-ethylphenol | 15.561 | 0.000219 | 3.6586 | 0.027178 |
| Cuminaldehyde | 15.225 | 0.000245 | 3.6108 | 0.027178 |
| SM 36:3;O2 | 15.116 | 0.000254 | 3.5951 | 0.027178 |
| 4-(2,5-Dimethylphenyl)-4-oxobutanoic acid | 14.896 | 0.000273 | 3.5633 | 0.027178 |
| Thymol | 13.96 | 0.000376 | 3.4242 | 0.032503 |
| 4-Prop-1-enylveratrole | 13.647 | 0.00042 | 3.3764 | 0.032503 |
| Propofol .beta.-D-glucuronide | 12.518 | 0.000634 | 3.1976 | 0.043441 |
| PPA | 12.308 | 0.000687 | 3.1633 | 0.043441 |
| Glu-Gly-Arg | 11.903 | 0.000801 | 3.0961 | 0.046486 |

**Table S4.2** Ascites Metabolic Signatures in OC II-III, OC IV, and GI Groups through ANOVA Post hoc analysis

| Metabolite | Group1 | Group2 | p | conf.low | conf.high | p.adj |
| --- | --- | --- | --- | --- | --- | --- |
| 2-tert-Butyl-4-ethylphenol | OC II-III | OC IV | 0 | 0.147 | 0.496 | 0.0414 |
| 2-tert-Butyl-4-ethylphenol | OC IV | GI | 0.3 | -0.226 | 1.025 | 0.304258 |
| 2-tert-Butyl-4-ethylphenol | OC II-III | GI | 0 | 0.256 | 0.758 | 0.004 |
| 3-Methoxy-4-(2-methylpropoxy)benzoic acid | OC II-III | OC IV | 0 | 0.25 | 0.961 | 0.0414 |
| 3-Methoxy-4-(2-methylpropoxy)benzoic acid | OC IV | GI | 0.8 | -0.688 | 0.621 | 0.780686 |
| 3-Methoxy-4-(2-methylpropoxy)benzoic acid | OC II-III | GI | 0 | 0.273 | 0.948 | 0.004 |
| 4-(2,5-Dimethylphenyl)-4-oxobutanoic acid | OC II-III | OC IV | 0.1 | -0.049 | 0.546 | 0.153692 |
| 4-(2,5-Dimethylphenyl)-4-oxobutanoic acid | OC IV | GI | 0.1 | -0.2 | 0.942 | 0.162 |
| 4-(2,5-Dimethylphenyl)-4-oxobutanoic acid | OC II-III | GI | 0 | 0.303 | 0.994 | 0.004 |
| 4-Isopropyl-3-methylphenol | OC II-III | OC IV | 0 | 0.293 | 0.753 | 0.0414 |
| 4-Isopropyl-3-methylphenol | OC IV | GI | 0.8 | -0.258 | 0.091 | 0.838 |
| 4-Isopropyl-3-methylphenol | OC II-III | GI | 0 | 0.331 | 0.564 | 0.004 |
| 4-Prop-1-enylveratrole | OC II-III | OC IV | 0 | 0.068 | 0.609 | 0.065455 |
| 4-Prop-1-enylveratrole | OC IV | GI | 0.3 | -0.438 | 0.439 | 0.34425 |
| 4-Prop-1-enylveratrole | OC II-III | GI | 0 | 0.19 | 0.616 | 0.009818 |
| Cuminaldehyde | OC II-III | OC IV | 0 | 0.071 | 0.874 | 0.0414 |
| Cuminaldehyde | OC IV | GI | 0.3 | -0.461 | 0.599 | 0.304258 |
| Cuminaldehyde | OC II-III | GI | 0 | 0.258 | 0.763 | 0.004 |
| Glu-Gly-Arg | OC II-III | OC IV | 0.2 | -0.014 | 0.34 | 0.212276 |
| Glu-Gly-Arg | OC IV | GI | 0.1 | -0.116 | 0.704 | 0.162 |
| Glu-Gly-Arg | OC II-III | GI | 0 | 0.129 | 0.761 | 0.004 |
| PPA | OC II-III | OC IV | 0 | 0.188 | 0.525 | 0.0414 |
| PPA | OC IV | GI | 0.5 | -0.273 | 0.897 | 0.502941 |
| PPA | OC II-III | GI | 0 | 0.201 | 1.124 | 0.004 |
| Phenylalanylphenylalanine | OC II-III | OC IV | 0 | -0.657 | -0.298 | 0.0414 |
| Phenylalanylphenylalanine | OC IV | GI | 0 | 0.135 | 0.484 | 0.0414 |
| Phenylalanylphenylalanine | OC II-III | GI | 0 | -0.253 | -0.029 | 0.067304 |
| Propofol .beta.-D-glucuronide | OC II-III | OC IV | 0 | 0.021 | 0.245 | 0.0414 |
| Propofol .beta.-D-glucuronide | OC IV | GI | 0.1 | -0.004 | 0.982 | 0.078 |
| Propofol .beta.-D-glucuronide | OC II-III | GI | 0 | 0.188 | 0.467 | 0.004 |
| SM 36:3;O2 | OC II-III | OC IV | 0.1 | -0.286 | 0.003 | 0.09792 |
| SM 36:3;O2 | OC IV | GI | 0 | 0.247 | 1.18 | 0.0414 |
| SM 36:3;O2 | OC II-III | GI | 0 | 0.16 | 0.822 | 0.009818 |
| Thymol | OC II-III | OC IV | 0 | 0.015 | 1.029 | 0.065455 |
| Thymol | OC IV | GI | 0.4 | -0.658 | 0.877 | 0.390545 |
| Thymol | OC II-III | GI | 0 | 0.292 | 1.016 | 0.004 |

**Table S5** Identification of Ascites Metabolic Signatures in OC and GI Groups.

| Metabolite | FC | log2(FC) | raw.pval | -LOG10(p) | p.ajusted |
| --- | --- | --- | --- | --- | --- |
| SM 36:3;O2 | 0.1777 | -2.4925 | 0.000107 | 3.97 | 0.051803 |
| Glu-Gly-Arg | 0.2232 | -2.1636 | 0.000258 | 3.5887 | 0.051803 |
| 4-(2,5-Dimethylphenyl)-4-oxobutanoic acid | 0.055543 | -4.1702 | 0.000268 | 3.5719 | 0.051803 |
| Propofol .beta.-D-glucuronide | 0.32339 | -1.6286 | 0.000298 | 3.5262 | 0.051803 |
| 4-Hydroxy-3-(3-methylbut-2-enyl)benzoic acid | 0.069655 | -3.8436 | 0.000803 | 3.0952 | 0.093227 |
| 2-tert-Butyl-4-ethylphenol | 0.10666 | -3.229 | 0.000911 | 3.0404 | 0.093227 |
| SM 18:2;O2/16:1 | 0.18188 | -2.4589 | 0.000938 | 3.028 | 0.093227 |
| Cyclo(leucylprolyl) | 2.7989 | 1.4849 | 0.001325 | 2.8778 | 0.11527 |
| Cefotaxime | 13.847 | 3.7915 | 0.001937 | 2.7129 | 0.14756 |
| SM 36:2;O2 | 0.3038 | -1.7188 | 0.00212 | 2.6737 | 0.14756 |
| Cuminaldehyde | 0.10596 | -3.2384 | 0.002496 | 2.6027 | 0.15612 |
| LPE 22:1 | 2.2785 | 1.1881 | 0.002882 | 2.5403 | 0.15612 |
| PPA | 0.16728 | -2.5796 | 0.003196 | 2.4954 | 0.15612 |
| 1-Methylguanine | 2.0611 | 1.0434 | 0.003354 | 2.4744 | 0.15612 |
| Thymol | 0.025805 | -5.2762 | 0.003636 | 2.4394 | 0.15612 |
| DG 36:3 | 0.40987 | -1.2868 | 0.003764 | 2.4243 | 0.15612 |
| 2-(4-Fluorophenyl)-N-(2-nitrobenzyl)ethanamine | 0.004909 | -7.6705 | 0.003921 | 2.4067 | 0.15612 |
| 3-Tert-Butyl-4-hydroxyanisole | 0.23819 | -2.0698 | 0.00418 | 2.3789 | 0.15612 |
| 3-Methoxy-4-(2-methylpropoxy)benzoic acid | 0.078529 | -3.6706 | 0.004273 | 2.3693 | 0.15612 |
| N-[3,5-bis(trifluoromethyl)phenyl]-6-oxo-1H-pyridazine-3-carboxamide | 8.5669 | 3.0988 | 0.00498 | 2.3027 | 0.15612 |
| Benzamide | 0.29756 | -1.7487 | 0.005207 | 2.2834 | 0.15612 |
| LPE 22:0 | 2.344 | 1.229 | 0.005552 | 2.2555 | 0.15612 |
| 3-(4-Isopropoxyphenyl)propanoic acid | 0.13777 | -2.8596 | 0.00576 | 2.2395 | 0.15612 |
| 4-Prop-1-enylveratrole | 0.23061 | -2.1165 | 0.006018 | 2.2205 | 0.15612 |
| SM 38:3;O2 | 0.40631 | -1.2994 | 0.006477 | 2.1886 | 0.15612 |
| CerP 30:0;O2 | 3.5215 | 1.8162 | 0.006675 | 2.1756 | 0.15612 |
| Cefdinir | 5.8359 | 2.545 | 0.00672 | 2.1727 | 0.15612 |
| 2,4-Diisopropylphenol | 0.60669 | -0.72096 | 0.006755 | 2.1704 | 0.15612 |
| DG 36:4 | 0.41777 | -1.2592 | 0.006768 | 2.1695 | 0.15612 |
| LPC 20:1 | 2.585 | 1.3702 | 0.00707 | 2.1506 | 0.15612 |
| 4-(Butylamino)benzoic acid | 1.5801 | 0.65997 | 0.007172 | 2.1444 | 0.15612 |
| 4-Isopropyl-3-methylphenol | 0.066962 | -3.9005 | 0.007178 | 2.144 | 0.15612 |
| SM 34:2;O2 | 0.2401 | -2.0583 | 0.007548 | 2.1222 | 0.15918 |
| SPB 16:0;O2 | 3.7683 | 1.9139 | 0.007817 | 2.107 | 0.16001 |
| 3-Methyl-1-adamantanecarboxylic acid | 0.32913 | -1.6033 | 0.008092 | 2.0919 | 0.16092 |
| CerP 28:2;O2 | 9.5671 | 3.2581 | 0.008477 | 2.0718 | 0.16388 |
| Caffeine | 8.951 | 3.1621 | 0.008857 | 2.0527 | 0.16661 |
| Glucosamine | 1.6169 | 0.69326 | 0.010106 | 1.9954 | 0.1849 |
| N'-Benzoyl-4-nitrobenzohydrazide | 0.29034 | -1.7842 | 0.010518 | 1.9781 | 0.1849 |
| Cefuroxime | 0.28769 | -1.7974 | 0.010755 | 1.9684 | 0.1849 |
| LPC 20:2 | 2.8778 | 1.525 | 0.010892 | 1.9629 | 0.1849 |
| Dexamethasone | 0.36553 | -1.4519 | 0.01196 | 1.9223 | 0.19819 |
| Levulinic acid | 2.4584 | 1.2977 | 0.014907 | 1.8266 | 0.23613 |
| Ethoxysulfuron | 0.2065 | -2.2758 | 0.014928 | 1.826 | 0.23613 |
| D-Tagatose | 13.211 | 3.7237 | 0.015826 | 1.8006 | 0.23686 |
| 3-Hydroxy-3-(nitromethyl)-1,3-dihydro-2H-indol-2-one | 0.26743 | -1.9028 | 0.01639 | 1.7854 | 0.23686 |
| 2-(1-Methylbutyl)phenol | 0.11859 | -3.076 | 0.016851 | 1.7734 | 0.23686 |
| Betamethasone 9,11-epoxide | 0.24994 | -2.0004 | 0.01725 | 1.7632 | 0.23686 |
| 1-(1Z-Hexadecenyl)-sn-glycero-3-phosphocholine | 5.4979 | 2.4589 | 0.017452 | 1.7582 | 0.23686 |
| 2-Pyrrolidinone | 2.5715 | 1.3626 | 0.017495 | 1.7571 | 0.23686 |
| Betamethasone | 0.16541 | -2.5959 | 0.017786 | 1.7499 | 0.23686 |
| 2-[(7-Amino-7H-[1,2,4]triazolo[4,3-b][1,2,4]triazol-3-yl)sulfanyl]-N-(2-pyrazinyl)acetamide | 0.1533 | -2.7056 | 0.017953 | 1.7459 | 0.23686 |
| (2E)-4-Oxo-4-[2-(3-pyridinylcarbonyl)hydrazino]-2-butenoic acid | 0.15586 | -2.6816 | 0.018419 | 1.7347 | 0.23686 |
| SM 38:2;O2 | 0.36853 | -1.4402 | 0.018534 | 1.732 | 0.23686 |
| 1,4-Dimethyl-2,6-dioxo-1,2,5,6-tetrahydropyridine-3-carbonitrile | 0.23758 | -2.0735 | 0.018718 | 1.7277 | 0.23686 |
| LPC 22:4 | 2.2465 | 1.1677 | 0.019258 | 1.7154 | 0.23934 |
| 3-Methylxanthine | 10.923 | 3.4493 | 0.020143 | 1.6959 | 0.24596 |
| 3,7-Dimethyluric acid | 6.0391 | 2.5943 | 0.021161 | 1.6745 | 0.253 |
| D-Glucurono-6,3-lactone | 2.1962 | 1.135 | 0.021652 | 1.6645 | 0.253 |
| SM 34:1;O2 | 0.37154 | -1.4284 | 0.02181 | 1.6613 | 0.253 |
| N-Acetyl-DL-valine | 2.0027 | 1.0019 | 0.02278 | 1.6424 | 0.25678 |
| Phosphocholine | 0.55701 | -0.84421 | 0.023198 | 1.6345 | 0.25678 |
| Remifentanil | 0.33739 | -1.5675 | 0.023243 | 1.6337 | 0.25678 |
| PC 40:6 | 0.57263 | -0.80432 | 0.024614 | 1.6088 | 0.26598 |
| LPE O-20:2 | 9.1866 | 3.1995 | 0.02484 | 1.6048 | 0.26598 |
| DGTS 36:1 | 0.48717 | -1.0375 | 0.026063 | 1.584 | 0.27394 |
| N-Phenyldiethanolamine | 0.15473 | -2.6921 | 0.02712 | 1.5667 | 0.27394 |
| 1,3-Dimethyluric acid | 4.3216 | 2.1116 | 0.027323 | 1.5635 | 0.27394 |
| CerP 32:0;O2 | 5.3248 | 2.4127 | 0.02744 | 1.5616 | 0.27394 |
| PC 38:6 | 0.46441 | -1.1065 | 0.027896 | 1.5545 | 0.27394 |
| Trimethylamine N-oxide | 1.8489 | 0.88671 | 0.027945 | 1.5537 | 0.27394 |
| SPB 20:1;O2 | 0.60845 | -0.71679 | 0.029391 | 1.5318 | 0.27879 |
| Glycerol tricaprylate | 0.46886 | -1.0928 | 0.029601 | 1.5287 | 0.27879 |
| Dodecylbenzenesulfonic acid | 1.7093 | 0.77342 | 0.030141 | 1.5208 | 0.27879 |
| CerP 30:1;O2 | 3.9252 | 1.9728 | 0.030383 | 1.5174 | 0.27879 |
| 3-Methylindole | 5.1711 | 2.3705 | 0.030443 | 1.5165 | 0.27879 |
| CerP 28:0;O2 | 3.1641 | 1.6618 | 0.030929 | 1.5096 | 0.27957 |
| Sordariol | 0.12568 | -2.9922 | 0.032885 | 1.483 | 0.29344 |
| CerP 32:1;O2 | 3.3157 | 1.7293 | 0.033322 | 1.4773 | 0.29357 |
| 1-(2-Hydroxyethyl)-2,2,6,6-tetramethyl-4-piperidinol | 14.902 | 3.8974 | 0.036673 | 1.4356 | 0.31543 |
| PE 40:6 | 0.5823 | -0.78018 | 0.036709 | 1.4352 | 0.31543 |
| Cer 42:1;O2 | 0.57398 | -0.80093 | 0.038908 | 1.41 | 0.33025 |
| CerP 34:1;O2 | 8.505 | 3.0883 | 0.039899 | 1.399 | 0.33458 |
| LPC 22:1 | 4.1677 | 2.0592 | 0.041927 | 1.3775 | 0.34739 |
| (9Z)-5,8,11-Trihydroxyoctadec-9-enoic acid | 29.45 | 4.8802 | 0.044126 | 1.3553 | 0.36131 |
| LPC 18:1 | 1.5505 | 0.63271 | 0.045051 | 1.3463 | 0.3646 |
| Diisopropanolamine | 0.098274 | -3.347 | 0.045639 | 1.3407 | 0.36511 |
| Z-Ligustilide | 0.11629 | -3.1042 | 0.046898 | 1.3288 | 0.37092 |
| Sphinganine | 0.18655 | -2.4224 | 0.047707 | 1.3214 | 0.37308 |
| 1-Dodecyl-2-pyrrolidinone | 2.1421 | 1.099 | 0.04898 | 1.31 | 0.37878 |

**Table S6** Identification of Ascites Metabolic Signatures in OC II-III and OC IV Groups.

| Metabolite | FC | log2(FC) | raw.pval | -LOG10(p) | p.ajusted |
| --- | --- | --- | --- | --- | --- |
| Phenylalanylphenylalanine | 4.3701 | 2.1277 | 3.86E-05 | 4.4137 | 0.025035 |
| 3-Methoxy-4-(2-methylpropoxy)benzoic acid | 0.050248 | -4.3148 | 0.000118 | 3.9278 | 0.031907 |
| PPA | 0.11044 | -3.1787 | 0.000147 | 3.8312 | 0.031907 |
| 2-(1-Methylbutyl)phenol | 0.047676 | -4.3906 | 0.000411 | 3.3866 | 0.061509 |
| Thioridazine | 0.044524 | -4.4893 | 0.000474 | 3.3243 | 0.061509 |
| Sordariol | 0.054121 | -4.2077 | 0.000833 | 3.0794 | 0.084447 |
| N.alpha.-Benzoyl-DL-arginine-4-nitroanilide | 7.2401 | 2.856 | 0.001144 | 2.9418 | 0.084447 |
| DL-2-hydroxy stearic acid | 3.0943 | 1.6296 | 0.001331 | 2.8757 | 0.084447 |
| Phe-Phe | 7.1625 | 2.8405 | 0.00137 | 2.8634 | 0.084447 |
| Naringenin | 0.092145 | -3.44 | 0.001429 | 2.8451 | 0.084447 |
| 4-Pyridoxic acid | 28.636 | 4.8398 | 0.001489 | 2.8272 | 0.084447 |
| 1-Methylhistidine | 4.891 | 2.2901 | 0.001624 | 2.7895 | 0.084447 |
| Quinolin-3-ol | 1.7355 | 0.79536 | 0.001692 | 2.7717 | 0.084447 |
| 3-(4-Isopropoxyphenyl)propanoic acid | 0.084253 | -3.5691 | 0.002074 | 2.6832 | 0.089854 |
| (1S,4R)-Bicyclo[2.2.1]hept-2-ylmethanamine | 0.35324 | -1.5013 | 0.002077 | 2.6826 | 0.089854 |
| Ropivacaine | 0.22001 | -2.1844 | 0.002357 | 2.6277 | 0.095591 |
| 2-tert-Butyl-4-ethylphenol | 0.12793 | -2.9665 | 0.002644 | 2.5777 | 0.10085 |
| Phenoxyacetate | 0.245 | -2.0292 | 0.002945 | 2.5309 | 0.10085 |
| o-Cresol | 0.193 | -2.3733 | 0.003065 | 2.5136 | 0.10085 |
| Phe Leu | 1.9588 | 0.96998 | 0.003108 | 2.5075 | 0.10085 |
| Stearamide | 0.5051 | -0.98535 | 0.003574 | 2.4468 | 0.11046 |
| 4-Isopropyl-3-methylphenol | 0.041857 | -4.5784 | 0.003759 | 2.4249 | 0.1109 |
| Benzyl alcohol | 0.085089 | -3.5549 | 0.004356 | 2.361 | 0.1205 |
| Fenuron | 0.025063 | -5.3183 | 0.004456 | 2.351 | 0.1205 |
| Indoleacrylic acid | 1.6511 | 0.72343 | 0.005389 | 2.2685 | 0.13797 |
| 2,5,7,8-Tetramethyl-2-(.beta.-carboxyethyl)-6-hydroxychroman | 0.35136 | -1.509 | 0.00553 | 2.2573 | 0.13797 |
| 3-Tert-Butyl-4-hydroxyanisole | 0.23281 | -2.1028 | 0.00574 | 2.2411 | 0.13797 |
| 16-Hydroxyhexadecanoic acid | 1.7895 | 0.83952 | 0.006359 | 2.1966 | 0.14434 |
| CerP 32:2;O2 | 1.6033 | 0.68109 | 0.00645 | 2.1905 | 0.14434 |
| 4-Prop-1-enylveratrole | 0.20767 | -2.2676 | 0.006764 | 2.1698 | 0.14633 |
| Azacyclotridecan-2-one | 0.21582 | -2.2121 | 0.007386 | 2.1316 | 0.15463 |
| Indole | 1.4219 | 0.50782 | 0.008449 | 2.0732 | 0.16809 |
| Phenol | 0.13383 | -2.9016 | 0.008563 | 2.0674 | 0.16809 |
| 4-Methyl-1-phenylpentan-3-one | 0.14046 | -2.8317 | 0.008806 | 2.0552 | 0.16809 |
| Thymol | 0.069031 | -3.8566 | 0.009917 | 2.0036 | 0.18352 |
| Octadecanedioic acid | 0.27826 | -1.8455 | 0.010239 | 1.9897 | 0.18352 |
| Cuminaldehyde | 0.16032 | -2.641 | 0.010463 | 1.9804 | 0.18352 |
| 2-Aminonaphthalene | 1.6178 | 0.69404 | 0.01311 | 1.8824 | 0.22391 |
| Propofol .beta.-D-glucuronide | 0.57238 | -0.80496 | 0.01491 | 1.8265 | 0.24257 |
| N-Acetyl-L-phenylalanine | 8.8978 | 3.1534 | 0.01495 | 1.8254 | 0.24257 |
| SM 34:1;O2 | 2.8656 | 1.5189 | 0.016469 | 1.7833 | 0.25507 |
| 3'-Hydroxyropivacaine | 0.20173 | -2.3095 | 0.016507 | 1.7823 | 0.25507 |
| PE O-38:5 | 0.20079 | -2.3162 | 0.017277 | 1.7625 | 0.26076 |
| PE O-36:5 | 0.16332 | -2.6142 | 0.017735 | 1.7512 | 0.2616 |
| N'-Benzoyl-4-nitrobenzohydrazide | 0.31187 | -1.681 | 0.018178 | 1.7405 | 0.26217 |
| 7-(4-fluoroanilino)-2-(2-morpholin-4-yl-2-oxoethyl)-[1,2,4]triazolo[4,3-a]pyrimidin-3-one | 0.15953 | -2.6481 | 0.018783 | 1.7262 | 0.26218 |
| Biliverdin | 2.0333 | 1.0238 | 0.018987 | 1.7215 | 0.26218 |
| SM 34:2;O2 | 3.0424 | 1.6052 | 0.019587 | 1.708 | 0.26483 |
| Benzocaine | 2.599 | 1.378 | 0.020243 | 1.6937 | 0.2659 |
| PELLETIERINE | 0.40023 | -1.3211 | 0.020485 | 1.6886 | 0.2659 |
| SM 38:2;O2 | 1.9834 | 0.98798 | 0.021836 | 1.6608 | 0.27389 |
| Cefuroxime | 0.3317 | -1.5921 | 0.021945 | 1.6587 | 0.27389 |
| SM 36:3;O2 | 2.1834 | 1.1266 | 0.022887 | 1.6404 | 0.28025 |
| ethyl 2-[3-(4-methylpiperidin-1-yl)sulfonyl-2-oxopyridin-1-yl]acetate | 0.34069 | -1.5535 | 0.023899 | 1.6216 | 0.28333 |
| Indole-3-carbinol | 1.5685 | 0.64943 | 0.024035 | 1.6192 | 0.28333 |
| LPC 22:1 | 5.6009 | 2.4857 | 0.025291 | 1.597 | 0.28333 |
| 6-Amino-2-benzoxazolinone | 0.096507 | -3.3732 | 0.025534 | 1.5929 | 0.28333 |
| Cholesterol sulfate | 0.31045 | -1.6876 | 0.025702 | 1.59 | 0.28333 |
| 2-Methyl-1-Pyrroline | 0.13094 | -2.933 | 0.025757 | 1.5891 | 0.28333 |
| Indoline | 2.3728 | 1.2466 | 0.026919 | 1.5699 | 0.28433 |
| Nudifloramide | 2.2696 | 1.1824 | 0.026953 | 1.5694 | 0.28433 |
| Remifentanil | 0.18219 | -2.4565 | 0.027528 | 1.5602 | 0.28433 |
| LPC 22:0 | 5.5003 | 2.4595 | 0.027925 | 1.554 | 0.28433 |
| Dexpanthenol | 21.055 | 4.3961 | 0.028081 | 1.5516 | 0.28433 |
| MG 14:4 | 2.9729 | 1.5719 | 0.028477 | 1.5455 | 0.28433 |
| 3-Hydroxyanthranilic acid | 2.2894 | 1.195 | 0.03137 | 1.5035 | 0.30847 |
| N-Acetyltryptophan | 4.0595 | 2.0213 | 0.032437 | 1.489 | 0.31068 |
| CerP 32:0;O2 | 6.0064 | 2.5865 | 0.03302 | 1.4812 | 0.31068 |
| N-(Tert-Butyl)-2-piperidinecarboxamide | 0.40872 | -1.2908 | 0.033031 | 1.4811 | 0.31068 |
| 3-Phenoxypropionic acid | 7.2324 | 2.8545 | 0.034418 | 1.4632 | 0.31911 |
| Hydroxypropionic acid | 4.5182 | 2.1758 | 0.036349 | 1.4395 | 0.32345 |
| PC O-38:5 | 2.0712 | 1.0504 | 0.036746 | 1.4348 | 0.32345 |
| 4-(2,5-Dimethylphenyl)-4-oxobutanoic acid | 0.18275 | -2.4521 | 0.037284 | 1.4285 | 0.32345 |
| LPI 18:1 | 1.5681 | 0.64906 | 0.037614 | 1.4247 | 0.32345 |
| 4-phenyl-1,5-diazecan-2-one | 0.14687 | -2.7674 | 0.037797 | 1.4225 | 0.32345 |
| Sulfolithocholic acid | 5.4611 | 2.4492 | 0.037878 | 1.4216 | 0.32345 |
| 3-Methyl-1-adamantanecarboxylic acid | 0.4171 | -1.2615 | 0.038819 | 1.411 | 0.32719 |
| 3,7-Dimethyl-2,6-octadienenitrile | 0.077861 | -3.683 | 0.04165 | 1.3804 | 0.34655 |
| 3-Hydroxy-3-(nitromethyl)-1,3-dihydro-2H-indol-2-one | 0.3495 | -1.5166 | 0.042706 | 1.3695 | 0.35013 |
| Mevalonic acid | 5.9764 | 2.5793 | 0.04316 | 1.3649 | 0.35013 |
| cis-4,10,13,16-Docosatetraenoic Acid | 1.9691 | 0.97757 | 0.044676 | 1.3499 | 0.35796 |
| Butyryl-L-carnitine | 3.837 | 1.94 | 0.046101 | 1.3363 | 0.36487 |
| Glu-Gly-Arg | 0.50365 | -0.98951 | 0.04897 | 1.3101 | 0.38074 |
| 13S-HOTrE(gamma) | 1.9485 | 0.96239 | 0.04928 | 1.3073 | 0.38074 |

**Table S7** Correlation Analysis Between Potential Microbiota-derived Metabolites and Flow Cytometry Cytokines/Chemokines between OC and GI

| Metabolite | Cytokine/Chemokine | pearson_correlation | p.value | adj.p.value | sig |
| --- | --- | --- | --- | --- | --- |
| Thymol | IL1B | 0.006971 | 0.9781 | 0.992016 | NA |
| Benzamide | IL1B | 0.454042 | 0.058392 | 0.472693 | NA |
| LPC 20:1 | IL1B | -0.12489 | 0.621463 | 0.992016 | NA |
| Caffeine | IL1B | -0.00626 | 0.980345 | 0.992016 | NA |
| Glucosamine | IL1B | -0.05477 | 0.829112 | 0.992016 | NA |
| LPC 20:2 | IL1B | -0.1229 | 0.627096 | 0.992016 | NA |
| Levulinic acid | IL1B | -0.15278 | 0.545023 | 0.980271 | NA |
| D-Tagatose | IL1B | -0.26614 | 0.285756 | 0.89117 | NA |
| LPC 22:4 | IL1B | 0.111396 | 0.659894 | 0.992016 | NA |
| 3-Methylxanthine | IL1B | -0.14072 | 0.577561 | 0.992016 | NA |
| D-Glucurono-6,3-lactone | IL1B | -0.21433 | 0.393088 | 0.937462 | NA |
| Phosphocholine | IL1B | 0.368237 | 0.132695 | 0.679945 | NA |
| Trimethylamine N-oxide | IL1B | 0.02287 | 0.928229 | 0.992016 | NA |
| 3-Methylindole | IL1B | -0.01854 | 0.941802 | 0.992016 | NA |
| LPC 22:1 | IL1B | -0.04277 | 0.866189 | 0.992016 | NA |
| LPC 18:1 | IL1B | -0.1025 | 0.685672 | 0.992016 | NA |
| Sphinganine | IL1B | -0.46221 | 0.053456 | 0.472693 | NA |
| Thymol | IFNa2 | -0.21235 | 0.397568 | 0.937462 | NA |
| Benzamide | IFNa2 | -0.23828 | 0.341008 | 0.920181 | NA |
| LPC 20:1 | IFNa2 | -0.0183 | 0.942541 | 0.992016 | NA |
| Caffeine | IFNa2 | 0.06919 | 0.785006 | 0.992016 | NA |
| Glucosamine | IFNa2 | 0.405164 | 0.095319 | 0.558766 | NA |
| LPC 20:2 | IFNa2 | 0.215496 | 0.390455 | 0.937462 | NA |
| Levulinic acid | IFNa2 | -0.15537 | 0.53816 | 0.980271 | NA |
| D-Tagatose | IFNa2 | 0.032342 | 0.898627 | 0.992016 | NA |
| LPC 22:4 | IFNa2 | 0.262689 | 0.292285 | 0.89117 | NA |
| 3-Methylxanthine | IFNa2 | 0.185143 | 0.462037 | 0.967673 | NA |
| D-Glucurono-6,3-lactone | IFNa2 | 0.639353 | 0.004278 | 0.248372 | ** |
| Phosphocholine | IFNa2 | -0.20566 | 0.412944 | 0.948655 | NA |
| Trimethylamine N-oxide | IFNa2 | -0.37265 | 0.127756 | 0.679945 | NA |
| 3-Methylindole | IFNa2 | -0.01638 | 0.948555 | 0.992016 | NA |
| LPC 22:1 | IFNa2 | 0.351314 | 0.15285 | 0.702283 | NA |
| LPC 18:1 | IFNa2 | 0.258799 | 0.299752 | 0.89117 | NA |
| Sphinganine | IFNa2 | -0.08042 | 0.751069 | 0.992016 | NA |
| Thymol | IFNr | 0.133034 | 0.598719 | 0.992016 | NA |
| Benzamide | IFNr | 0.05384 | 0.831968 | 0.992016 | NA |
| LPC 20:1 | IFNr | -0.33003 | 0.18106 | 0.73756 | NA |
| Caffeine | IFNr | -0.09792 | 0.699079 | 0.992016 | NA |
| Glucosamine | IFNr | 0.089857 | 0.722903 | 0.992016 | NA |
| LPC 20:2 | IFNr | -0.04463 | 0.860406 | 0.992016 | NA |
| Levulinic acid | IFNr | -0.47558 | 0.046067 | 0.472693 | * |
| D-Tagatose | IFNr | -0.1681 | 0.504931 | 0.980271 | NA |
| LPC 22:4 | IFNr | -0.04003 | 0.874705 | 0.992016 | NA |
| 3-Methylxanthine | IFNr | -0.09185 | 0.716989 | 0.992016 | NA |
| D-Glucurono-6,3-lactone | IFNr | 0.363156 | 0.13854 | 0.679945 | NA |
| Phosphocholine | IFNr | 0.03202 | 0.899631 | 0.992016 | NA |
| Trimethylamine N-oxide | IFNr | -0.52848 | 0.024153 | 0.472693 | * |
| 3-Methylindole | IFNr | -0.2245 | 0.370455 | 0.928826 | NA |
| LPC 22:1 | IFNr | 0.011482 | 0.963935 | 0.992016 | NA |
| LPC 18:1 | IFNr | 0.041826 | 0.869113 | 0.992016 | NA |
| Sphinganine | IFNr | 0.152 | 0.547108 | 0.980271 | NA |
| Thymol | TNFa | -0.13097 | 0.604458 | 0.992016 | NA |
| Benzamide | TNFa | -0.18154 | 0.470936 | 0.967673 | NA |
| LPC 20:1 | TNFa | -0.26239 | 0.292852 | 0.89117 | NA |
| Caffeine | TNFa | 0.067517 | 0.790094 | 0.992016 | NA |
| Glucosamine | TNFa | 0.29937 | 0.227487 | 0.808919 | NA |
| LPC 20:2 | TNFa | -0.04312 | 0.865092 | 0.992016 | NA |
| Levulinic acid | TNFa | -0.21375 | 0.394397 | 0.937462 | NA |
| D-Tagatose | TNFa | 0.015632 | 0.950911 | 0.992016 | NA |
| LPC 22:4 | TNFa | 0.073134 | 0.773047 | 0.992016 | NA |
| 3-Methylxanthine | TNFa | 0.029815 | 0.906511 | 0.992016 | NA |
| D-Glucurono-6,3-lactone | TNFa | 0.490201 | 0.038901 | 0.472693 | * |
| Phosphocholine | TNFa | -0.09166 | 0.717563 | 0.992016 | NA |
| Trimethylamine N-oxide | TNFa | -0.3708 | 0.129813 | 0.679945 | NA |
| 3-Methylindole | TNFa | -0.01657 | 0.947954 | 0.992016 | NA |
| LPC 22:1 | TNFa | 0.243237 | 0.330756 | 0.906911 | NA |
| LPC 18:1 | TNFa | -0.0267 | 0.91625 | 0.992016 | NA |
| Sphinganine | TNFa | -0.03937 | 0.876741 | 0.992016 | NA |
| Thymol | MCP1 | 0.159141 | 0.528208 | 0.980271 | NA |
| Benzamide | MCP1 | 0.224013 | 0.37153 | 0.928826 | NA |
| LPC 20:1 | MCP1 | -0.42488 | 0.078815 | 0.498703 | NA |
| Caffeine | MCP1 | -0.33754 | 0.170726 | 0.725587 | NA |
| Glucosamine | MCP1 | -0.35153 | 0.152582 | 0.702283 | NA |
| LPC 20:2 | MCP1 | -0.4616 | 0.053813 | 0.472693 | NA |
| Levulinic acid | MCP1 | 0.021273 | 0.93323 | 0.992016 | NA |
| D-Tagatose | MCP1 | -0.5931 | 0.009477 | 0.402771 | ** |
| LPC 22:4 | MCP1 | -0.43235 | 0.07315 | 0.498703 | NA |
| 3-Methylxanthine | MCP1 | -0.30499 | 0.21846 | 0.807353 | NA |
| D-Glucurono-6,3-lactone | MCP1 | -0.25391 | 0.309288 | 0.89117 | NA |
| Phosphocholine | MCP1 | -0.08792 | 0.728649 | 0.992016 | NA |
| Trimethylamine N-oxide | MCP1 | -0.00021 | 0.999348 | 0.999348 | NA |
| 3-Methylindole | MCP1 | -0.25921 | 0.298959 | 0.89117 | NA |
| LPC 22:1 | MCP1 | -0.32317 | 0.190843 | 0.73756 | NA |
| LPC 18:1 | MCP1 | -0.38381 | 0.115856 | 0.656516 | NA |
| Sphinganine | MCP1 | 0.069918 | 0.782796 | 0.992016 | NA |
| Thymol | IL6 | -0.2466 | 0.323912 | 0.902706 | NA |
| Benzamide | IL6 | -0.01662 | 0.947814 | 0.992016 | NA |
| LPC 20:1 | IL6 | 0.228102 | 0.362632 | 0.928826 | NA |
| Caffeine | IL6 | 0.001547 | 0.995138 | 0.999348 | NA |
| Glucosamine | IL6 | 0.256183 | 0.304837 | 0.89117 | NA |
| LPC 20:2 | IL6 | 0.137262 | 0.587041 | 0.992016 | NA |
| Levulinic acid | IL6 | 0.198014 | 0.430909 | 0.951358 | NA |
| D-Tagatose | IL6 | -0.11265 | 0.656287 | 0.992016 | NA |
| LPC 22:4 | IL6 | 0.424382 | 0.079206 | 0.498703 | NA |
| 3-Methylxanthine | IL6 | 0.199719 | 0.426869 | 0.951358 | NA |
| D-Glucurono-6,3-lactone | IL6 | 0.022497 | 0.929397 | 0.992016 | NA |
| Phosphocholine | IL6 | -0.1284 | 0.611622 | 0.992016 | NA |
| Trimethylamine N-oxide | IL6 | 0.298809 | 0.228401 | 0.808919 | NA |
| 3-Methylindole | IL6 | 0.102057 | 0.686978 | 0.992016 | NA |
| LPC 22:1 | IL6 | 0.072969 | 0.773545 | 0.992016 | NA |
| LPC 18:1 | IL6 | 0.086564 | 0.732701 | 0.992016 | NA |
| Sphinganine | IL6 | -0.27537 | 0.268728 | 0.89117 | NA |
| Thymol | IL8 | -0.17763 | 0.480718 | 0.972881 | NA |
| Benzamide | IL8 | -0.08518 | 0.736826 | 0.992016 | NA |
| LPC 20:1 | IL8 | -0.274 | 0.271215 | 0.89117 | NA |
| Caffeine | IL8 | 0.028407 | 0.910909 | 0.992016 | NA |
| Glucosamine | IL8 | -0.02664 | 0.916435 | 0.992016 | NA |
| LPC 20:2 | IL8 | -0.32313 | 0.190898 | 0.73756 | NA |
| Levulinic acid | IL8 | 0.098721 | 0.696741 | 0.992016 | NA |
| D-Tagatose | IL8 | -0.03426 | 0.892649 | 0.992016 | NA |
| LPC 22:4 | IL8 | -0.10789 | 0.670021 | 0.992016 | NA |
| 3-Methylxanthine | IL8 | 0.011987 | 0.962347 | 0.992016 | NA |
| D-Glucurono-6,3-lactone | IL8 | 0.034115 | 0.893099 | 0.992016 | NA |
| Phosphocholine | IL8 | -0.1426 | 0.572439 | 0.992016 | NA |
| Trimethylamine N-oxide | IL8 | 0.088293 | 0.727551 | 0.992016 | NA |
| 3-Methylindole | IL8 | -0.1178 | 0.641549 | 0.992016 | NA |
| LPC 22:1 | IL8 | -0.01283 | 0.959709 | 0.992016 | NA |
| LPC 18:1 | IL8 | -0.30778 | 0.214051 | 0.807353 | NA |
| Sphinganine | IL8 | -0.13547 | 0.59198 | 0.992016 | NA |
| Thymol | IL10 | -0.47129 | 0.048349 | 0.472693 | * |
| Benzamide | IL10 | -0.48586 | 0.040931 | 0.472693 | * |
| LPC 20:1 | IL10 | 0.496278 | 0.036188 | 0.472693 | * |
| Caffeine | IL10 | 0.488672 | 0.039608 | 0.472693 | * |
| Glucosamine | IL10 | 0.704975 | 0.001085 | 0.184532 | ** |
| LPC 20:2 | IL10 | 0.341126 | 0.165947 | 0.723357 | NA |
| Levulinic acid | IL10 | 0.430391 | 0.074605 | 0.498703 | NA |
| D-Tagatose | IL10 | 0.638046 | 0.004383 | 0.248372 | ** |
| LPC 22:4 | IL10 | 0.548344 | 0.018462 | 0.472693 | * |
| 3-Methylxanthine | IL10 | 0.459994 | 0.054763 | 0.472693 | NA |
| D-Glucurono-6,3-lactone | IL10 | 0.260534 | 0.296407 | 0.89117 | NA |
| Phosphocholine | IL10 | -0.32445 | 0.188988 | 0.73756 | NA |
| Trimethylamine N-oxide | IL10 | 0.481896 | 0.042858 | 0.472693 | * |
| 3-Methylindole | IL10 | 0.347593 | 0.157548 | 0.704818 | NA |
| LPC 22:1 | IL10 | 0.449642 | 0.061187 | 0.472806 | NA |
| LPC 18:1 | IL10 | 0.201551 | 0.422552 | 0.951358 | NA |
| Sphinganine | IL10 | -0.23292 | 0.352292 | 0.928826 | NA |
| Thymol | IL18 | 0.168453 | 0.504019 | 0.980271 | NA |
| Benzamide | IL18 | 0.151741 | 0.547798 | 0.980271 | NA |
| LPC 20:1 | IL18 | 0.006421 | 0.979826 | 0.992016 | NA |
| Caffeine | IL18 | -0.47537 | 0.046178 | 0.472693 | * |
| Glucosamine | IL18 | -0.10178 | 0.687786 | 0.992016 | NA |
| LPC 20:2 | IL18 | 0.190393 | 0.449204 | 0.966642 | NA |
| Levulinic acid | IL18 | -0.22964 | 0.359306 | 0.928826 | NA |
| D-Tagatose | IL18 | -0.36192 | 0.139989 | 0.679945 | NA |
| LPC 22:4 | IL18 | 0.081967 | 0.74644 | 0.992016 | NA |
| 3-Methylxanthine | IL18 | -0.21017 | 0.402557 | 0.937462 | NA |
| D-Glucurono-6,3-lactone | IL18 | 0.285061 | 0.251558 | 0.872751 | NA |
| Phosphocholine | IL18 | 0.023709 | 0.925602 | 0.992016 | NA |
| Trimethylamine N-oxide | IL18 | -0.42026 | 0.082484 | 0.500795 | NA |
| 3-Methylindole | IL18 | 0.045926 | 0.856404 | 0.992016 | NA |
| LPC 22:1 | IL18 | -0.15531 | 0.53831 | 0.980271 | NA |
| LPC 18:1 | IL18 | 0.167963 | 0.505281 | 0.980271 | NA |
| Sphinganine | IL18 | 0.454801 | 0.057919 | 0.472693 | NA |
| Thymol | IL23 | -0.03218 | 0.89913 | 0.992016 | NA |
| Benzamide | IL23 | -0.06572 | 0.795557 | 0.992016 | NA |
| LPC 20:1 | IL23 | -0.18093 | 0.472452 | 0.967673 | NA |
| Caffeine | IL23 | -0.01828 | 0.942612 | 0.992016 | NA |
| Glucosamine | IL23 | 0.250592 | 0.315876 | 0.894982 | NA |
| LPC 20:2 | IL23 | 0.114099 | 0.652129 | 0.992016 | NA |
| Levulinic acid | IL23 | -0.42684 | 0.077296 | 0.498703 | NA |
| D-Tagatose | IL23 | -0.0969 | 0.7021 | 0.992016 | NA |
| LPC 22:4 | IL23 | 0.15622 | 0.535901 | 0.980271 | NA |
| 3-Methylxanthine | IL23 | 0.082111 | 0.746009 | 0.992016 | NA |
| D-Glucurono-6,3-lactone | IL23 | 0.487065 | 0.040361 | 0.472693 | * |
| Phosphocholine | IL23 | -0.04447 | 0.860927 | 0.992016 | NA |
| Trimethylamine N-oxide | IL23 | -0.4909 | 0.038584 | 0.472693 | * |
| 3-Methylindole | IL23 | -0.15203 | 0.54702 | 0.980271 | NA |
| LPC 22:1 | IL23 | 0.182524 | 0.468504 | 0.967673 | NA |
| LPC 18:1 | IL23 | 0.192905 | 0.443131 | 0.965798 | NA |
| Sphinganine | IL23 | -0.01452 | 0.954386 | 0.992016 | NA |

**Table S8** Correlation Analysis Between Potential Microbiota-derived Metabolites and Flow Cytometry Cytokines/Chemokines between OC II-III and OC IV

| Metabolite | Cytokine/Chemokine | pearson_correlation | p.value | adj.p.value | sig |
| --- | --- | --- | --- | --- | --- |
| Naringenin | IL1B | -0.82303 | 0.003445 | 0.161669 | ** |
| 4-Pyridoxic acid | IL1B | 0.76393 | 0.010104 | 0.161669 | * |
| 1-Methylhistidine | IL1B | 0.651744 | 0.041168 | 0.299404 | * |
| o-Cresol | IL1B | -0.79362 | 0.006135 | 0.161669 | ** |
| Benzyl alcohol | IL1B | -0.66315 | 0.036601 | 0.299404 | * |
| Indole | IL1B | 0.352278 | 0.318113 | 0.547292 | NA |
| Phenol | IL1B | -0.6661 | 0.03548 | 0.299404 | * |
| Octadecanedioic acid | IL1B | -0.81963 | 0.003702 | 0.161669 | ** |
| N-Acetyl-L-phenylalanine | IL1B | 0.600047 | 0.066661 | 0.395028 | NA |
| Biliverdin | IL1B | 0.548237 | 0.100836 | 0.44034 | NA |
| Nudifloramide | IL1B | 0.47609 | 0.164238 | 0.45992 | NA |
| 3-Hydroxyanthranilic acid | IL1B | 0.433829 | 0.210343 | 0.467429 | NA |
| Hydroxypropionic acid | IL1B | 0.54511 | 0.103193 | 0.44034 | NA |
| LPI 18:1 | IL1B | 0.698338 | 0.024692 | 0.299404 | * |
| Mevalonic acid | IL1B | 0.655189 | 0.039751 | 0.299404 | * |
| Butyryl-L-carnitine | IL1B | 0.479856 | 0.160456 | 0.458444 | NA |
| Naringenin | IFNa2 | -0.45964 | 0.181391 | 0.460675 | NA |
| 4-Pyridoxic acid | IFNa2 | 0.571481 | 0.084379 | 0.431318 | NA |
| 1-Methylhistidine | IFNa2 | 0.439702 | 0.203535 | 0.467429 | NA |
| o-Cresol | IFNa2 | -0.21707 | 0.546913 | 0.717264 | NA |
| Benzyl alcohol | IFNa2 | -0.15559 | 0.667758 | 0.766842 | NA |
| Indole | IFNa2 | 0.37317 | 0.288183 | 0.512325 | NA |
| Phenol | IFNa2 | -0.08965 | 0.805454 | 0.843788 | NA |
| Octadecanedioic acid | IFNa2 | -0.34232 | 0.332927 | 0.554727 | NA |
| N-Acetyl-L-phenylalanine | IFNa2 | 0.56474 | 0.088959 | 0.431318 | NA |
| Biliverdin | IFNa2 | 0.626044 | 0.052827 | 0.338091 | NA |
| Nudifloramide | IFNa2 | 0.219312 | 0.542671 | 0.717264 | NA |
| 3-Hydroxyanthranilic acid | IFNa2 | 0.182759 | 0.613303 | 0.735614 | NA |
| Hydroxypropionic acid | IFNa2 | 0.518581 | 0.1246 | 0.453091 | NA |
| LPI 18:1 | IFNa2 | -0.07924 | 0.827744 | 0.859994 | NA |
| Mevalonic acid | IFNa2 | 0.192574 | 0.594022 | 0.734622 | NA |
| Butyryl-L-carnitine | IFNa2 | 0.512321 | 0.130022 | 0.458444 | NA |
| Naringenin | IFNr | -0.39003 | 0.265185 | 0.499399 | NA |
| 4-Pyridoxic acid | IFNr | 0.444527 | 0.198038 | 0.467429 | NA |
| 1-Methylhistidine | IFNr | 0.377082 | 0.282754 | 0.508321 | NA |
| o-Cresol | IFNr | -0.21121 | 0.558036 | 0.720046 | NA |
| Benzyl alcohol | IFNr | -0.15428 | 0.670433 | 0.766842 | NA |
| Indole | IFNr | 0.238297 | 0.507332 | 0.699768 | NA |
| Phenol | IFNr | -0.1033 | 0.77643 | 0.823352 | NA |
| Octadecanedioic acid | IFNr | -0.40136 | 0.250314 | 0.489138 | NA |
| N-Acetyl-L-phenylalanine | IFNr | 0.525707 | 0.118602 | 0.451815 | NA |
| Biliverdin | IFNr | 0.470256 | 0.170205 | 0.45992 | NA |
| Nudifloramide | IFNr | 0.208631 | 0.562971 | 0.720603 | NA |
| 3-Hydroxyanthranilic acid | IFNr | 0.115164 | 0.751393 | 0.823352 | NA |
| Hydroxypropionic acid | IFNr | 0.357355 | 0.310697 | 0.545794 | NA |
| LPI 18:1 | IFNr | -0.06094 | 0.867197 | 0.892828 | NA |
| Mevalonic acid | IFNr | 0.188768 | 0.601472 | 0.734622 | NA |
| Butyryl-L-carnitine | IFNr | 0.434132 | 0.209988 | 0.467429 | NA |
| Naringenin | TNFa | -0.46604 | 0.174594 | 0.45992 | NA |
| 4-Pyridoxic acid | TNFa | 0.547532 | 0.101364 | 0.44034 | NA |
| 1-Methylhistidine | TNFa | 0.435655 | 0.208212 | 0.467429 | NA |
| o-Cresol | TNFa | -0.24302 | 0.49869 | 0.697577 | NA |
| Benzyl alcohol | TNFa | -0.18136 | 0.616077 | 0.735614 | NA |
| Indole | TNFa | 0.337571 | 0.340114 | 0.555288 | NA |
| Phenol | TNFa | -0.12248 | 0.736064 | 0.812209 | NA |
| Octadecanedioic acid | TNFa | -0.38696 | 0.269295 | 0.499399 | NA |
| N-Acetyl-L-phenylalanine | TNFa | 0.568334 | 0.086498 | 0.431318 | NA |
| Biliverdin | TNFa | 0.575874 | 0.081476 | 0.431318 | NA |
| Nudifloramide | TNFa | 0.214149 | 0.552448 | 0.718631 | NA |
| 3-Hydroxyanthranilic acid | TNFa | 0.163956 | 0.650832 | 0.760096 | NA |
| Hydroxypropionic acid | TNFa | 0.484078 | 0.156277 | 0.458444 | NA |
| LPI 18:1 | TNFa | -0.05438 | 0.881394 | 0.892828 | NA |
| Mevalonic acid | TNFa | 0.21856 | 0.544091 | 0.717264 | NA |
| Butyryl-L-carnitine | TNFa | 0.491089 | 0.149484 | 0.458444 | NA |
| Naringenin | MCP1 | 0.680559 | 0.030311 | 0.299404 | * |
| 4-Pyridoxic acid | MCP1 | -0.64343 | 0.04473 | 0.311165 | * |
| 1-Methylhistidine | MCP1 | -0.73926 | 0.014556 | 0.21172 | * |
| o-Cresol | MCP1 | 0.771495 | 0.008958 | 0.161669 | ** |
| Benzyl alcohol | MCP1 | 0.765502 | 0.009858 | 0.161669 | ** |
| Indole | MCP1 | -0.53962 | 0.107415 | 0.44034 | NA |
| Phenol | MCP1 | 0.770968 | 0.009035 | 0.161669 | ** |
| Octadecanedioic acid | MCP1 | 0.764992 | 0.009938 | 0.161669 | ** |
| N-Acetyl-L-phenylalanine | MCP1 | -0.58503 | 0.07564 | 0.417325 | NA |
| Biliverdin | MCP1 | -0.46184 | 0.179041 | 0.460675 | NA |
| Nudifloramide | MCP1 | -0.46844 | 0.172089 | 0.45992 | NA |
| 3-Hydroxyanthranilic acid | MCP1 | -0.32008 | 0.367265 | 0.576101 | NA |
| Hydroxypropionic acid | MCP1 | -0.55522 | 0.095696 | 0.44034 | NA |
| LPI 18:1 | MCP1 | -0.26475 | 0.459766 | 0.668893 | NA |
| Mevalonic acid | MCP1 | -0.8718 | 0.00101 | 0.161526 | ** |
| Butyryl-L-carnitine | MCP1 | -0.53568 | 0.110511 | 0.44034 | NA |
| Naringenin | IL6 | 0.434197 | 0.209913 | 0.467429 | NA |
| 4-Pyridoxic acid | IL6 | -0.50331 | 0.138079 | 0.458444 | NA |
| 1-Methylhistidine | IL6 | -0.50783 | 0.134 | 0.458444 | NA |
| o-Cresol | IL6 | 0.355202 | 0.313831 | 0.545794 | NA |
| Benzyl alcohol | IL6 | 0.465327 | 0.175345 | 0.45992 | NA |
| Indole | IL6 | -0.69116 | 0.026867 | 0.299404 | * |
| Phenol | IL6 | 0.385773 | 0.270892 | 0.499399 | NA |
| Octadecanedioic acid | IL6 | 0.349203 | 0.322651 | 0.549193 | NA |
| N-Acetyl-L-phenylalanine | IL6 | -0.65506 | 0.039804 | 0.299404 | * |
| Biliverdin | IL6 | -0.54249 | 0.105196 | 0.44034 | NA |
| Nudifloramide | IL6 | -0.66246 | 0.036867 | 0.299404 | * |
| 3-Hydroxyanthranilic acid | IL6 | -0.62711 | 0.052304 | 0.338091 | NA |
| Hydroxypropionic acid | IL6 | -0.44522 | 0.197259 | 0.467429 | NA |
| LPI 18:1 | IL6 | -0.08899 | 0.806872 | 0.843788 | NA |
| Mevalonic acid | IL6 | -0.2657 | 0.4581 | 0.668893 | NA |
| Butyryl-L-carnitine | IL6 | -0.66397 | 0.036288 | 0.299404 | * |
| Naringenin | IL8 | 0.404661 | 0.246069 | 0.489138 | NA |
| 4-Pyridoxic acid | IL8 | -0.31059 | 0.382412 | 0.588327 | NA |
| 1-Methylhistidine | IL8 | -0.22271 | 0.536273 | 0.717264 | NA |
| o-Cresol | IL8 | 0.402947 | 0.248267 | 0.489138 | NA |
| Benzyl alcohol | IL8 | 0.486264 | 0.154138 | 0.458444 | NA |
| Indole | IL8 | -0.2647 | 0.459864 | 0.668893 | NA |
| Phenol | IL8 | 0.448303 | 0.193797 | 0.467429 | NA |
| Octadecanedioic acid | IL8 | 0.256234 | 0.474862 | 0.684486 | NA |
| N-Acetyl-L-phenylalanine | IL8 | -0.49686 | 0.144032 | 0.458444 | NA |
| Biliverdin | IL8 | -0.18913 | 0.600762 | 0.734622 | NA |
| Nudifloramide | IL8 | -0.69312 | 0.026259 | 0.299404 | * |
| 3-Hydroxyanthranilic acid | IL8 | -0.7871 | 0.00689 | 0.161669 | ** |
| Hydroxypropionic acid | IL8 | -0.05653 | 0.876742 | 0.892828 | NA |
| LPI 18:1 | IL8 | -0.62098 | 0.055356 | 0.340653 | NA |
| Mevalonic acid | IL8 | -0.05281 | 0.88479 | 0.892828 | NA |
| Butyryl-L-carnitine | IL8 | -0.5219 | 0.121782 | 0.453091 | NA |
| Naringenin | IL10 | 0.206227 | 0.567579 | 0.720736 | NA |
| 4-Pyridoxic acid | IL10 | -0.24797 | 0.489713 | 0.693398 | NA |
| 1-Methylhistidine | IL10 | 0.106424 | 0.769816 | 0.823352 | NA |
| o-Cresol | IL10 | -0.127 | 0.726621 | 0.811546 | NA |
| Benzyl alcohol | IL10 | -0.10571 | 0.771336 | 0.823352 | NA |
| Indole | IL10 | -0.23491 | 0.513559 | 0.702304 | NA |
| Phenol | IL10 | -0.20058 | 0.578465 | 0.723081 | NA |
| Octadecanedioic acid | IL10 | -0.13673 | 0.706432 | 0.79598 | NA |
| N-Acetyl-L-phenylalanine | IL10 | -0.41673 | 0.230899 | 0.486104 | NA |
| Biliverdin | IL10 | -0.34247 | 0.332706 | 0.554727 | NA |
| Nudifloramide | IL10 | -0.28586 | 0.423324 | 0.645065 | NA |
| 3-Hydroxyanthranilic acid | IL10 | -0.50165 | 0.139599 | 0.458444 | NA |
| Hydroxypropionic acid | IL10 | -0.22681 | 0.528606 | 0.716753 | NA |
| LPI 18:1 | IL10 | -0.10566 | 0.771428 | 0.823352 | NA |
| Mevalonic acid | IL10 | 0.421872 | 0.224604 | 0.480662 | NA |
| Butyryl-L-carnitine | IL10 | -0.38529 | 0.271548 | 0.499399 | NA |
| Naringenin | IL18 | 0.314602 | 0.375972 | 0.584034 | NA |
| 4-Pyridoxic acid | IL18 | -0.26989 | 0.450772 | 0.668893 | NA |
| 1-Methylhistidine | IL18 | -0.32117 | 0.365531 | 0.576101 | NA |
| o-Cresol | IL18 | 0.403195 | 0.247949 | 0.489138 | NA |
| Benzyl alcohol | IL18 | 0.396169 | 0.257066 | 0.495549 | NA |
| Indole | IL18 | -0.32492 | 0.359636 | 0.575417 | NA |
| Phenol | IL18 | 0.403566 | 0.247472 | 0.489138 | NA |
| Octadecanedioic acid | IL18 | 0.588013 | 0.073796 | 0.417325 | NA |
| N-Acetyl-L-phenylalanine | IL18 | -0.27156 | 0.447873 | 0.668893 | NA |
| Biliverdin | IL18 | -0.17302 | 0.632642 | 0.744284 | NA |
| Nudifloramide | IL18 | -0.37825 | 0.28115 | 0.508321 | NA |
| 3-Hydroxyanthranilic acid | IL18 | -0.24155 | 0.501383 | 0.697577 | NA |
| Hydroxypropionic acid | IL18 | -0.33443 | 0.344909 | 0.557429 | NA |
| LPI 18:1 | IL18 | -0.25363 | 0.479527 | 0.685038 | NA |
| Mevalonic acid | IL18 | -0.4846 | 0.155768 | 0.458444 | NA |
| Butyryl-L-carnitine | IL18 | -0.12519 | 0.730392 | 0.811546 | NA |
| Naringenin | IL23 | -0.42129 | 0.22531 | 0.480662 | NA |
| 4-Pyridoxic acid | IL23 | 0.483735 | 0.156614 | 0.458444 | NA |
| 1-Methylhistidine | IL23 | 0.340083 | 0.336303 | 0.554727 | NA |
| o-Cresol | IL23 | -0.17681 | 0.625094 | 0.740852 | NA |
| Benzyl alcohol | IL23 | -0.10301 | 0.777038 | 0.823352 | NA |
| Indole | IL23 | 0.183208 | 0.612415 | 0.735614 | NA |
| Phenol | IL23 | -0.05168 | 0.887247 | 0.892828 | NA |
| Octadecanedioic acid | IL23 | -0.40107 | 0.250683 | 0.489138 | NA |
| N-Acetyl-L-phenylalanine | IL23 | 0.53277 | 0.112837 | 0.44034 | NA |
| Biliverdin | IL23 | 0.481199 | 0.159119 | 0.458444 | NA |
| Nudifloramide | IL23 | 0.201261 | 0.577145 | 0.723081 | NA |
| 3-Hydroxyanthranilic acid | IL23 | 0.154007 | 0.670986 | 0.766842 | NA |
| Hydroxypropionic acid | IL23 | 0.450643 | 0.191197 | 0.467429 | NA |
| LPI 18:1 | IL23 | -0.04045 | 0.911664 | 0.911664 | NA |
| Mevalonic acid | IL23 | 0.150798 | 0.677528 | 0.768826 | NA |
| Butyryl-L-carnitine | IL23 | 0.429483 | 0.215465 | 0.472251 | NA |


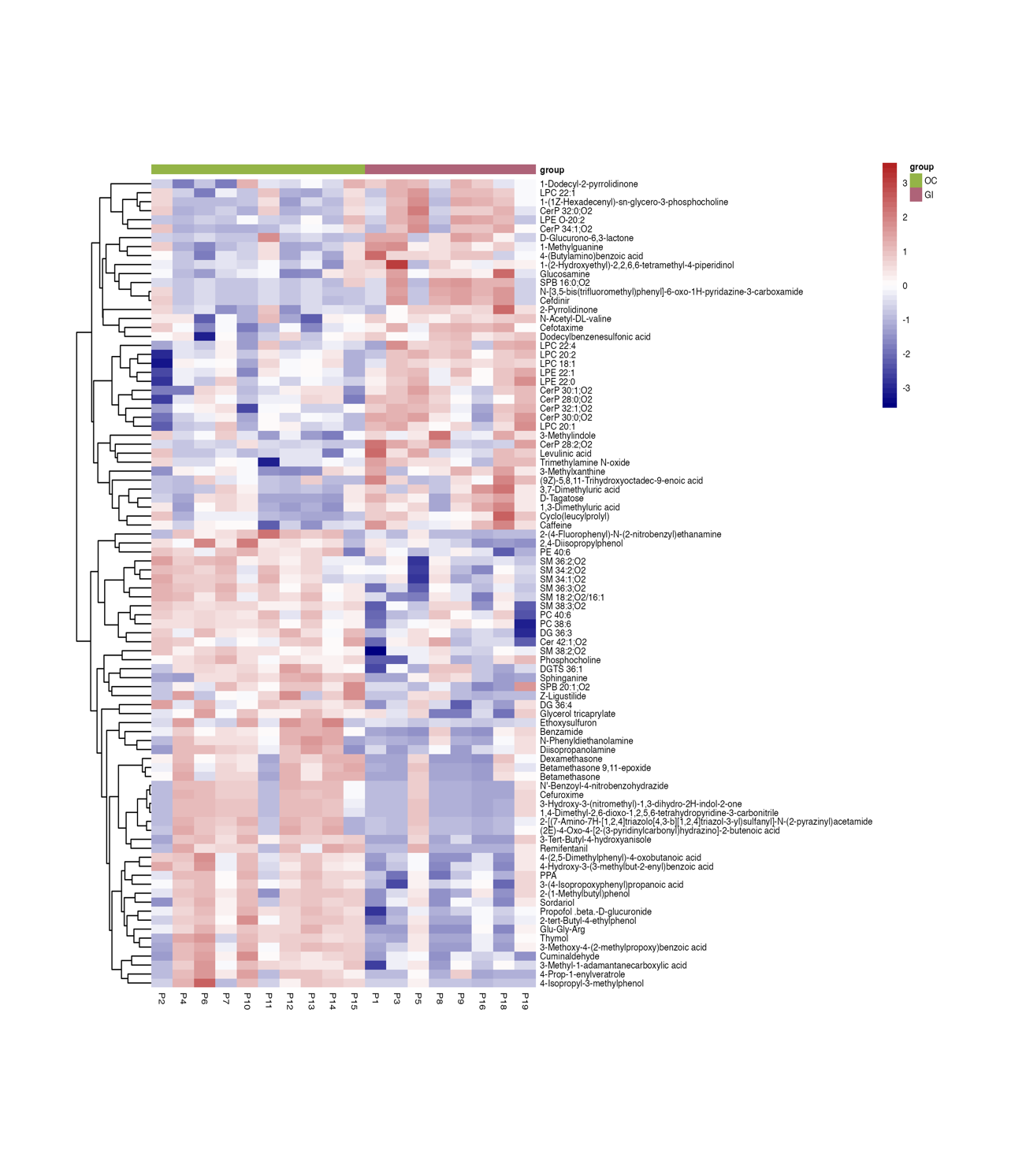


***Figure S1. Identification of Ascites Metabolic Signatures in OC and GI Groups.***

*The heatmap displays 90 significant metabolites (t-test, raw p-value cutoff of 0.05, fold change threshold of 1.2) across ten OC and eight GI biological replicates. Clustering was performed using Ward’s hierarchical method with Euclidean distance as the distance metric. Abbreviations: LPC, lysophosphatidylcholine; CerP, Ceramide phosphate; LPE, lysophosphatidylethanolamine; PE, Phosphatidylethanolamine; SPB, Sphingoid base; SM, sphingomyelin; PC, Phosphatidylcholine; DG, Diacylglycerol; Cer, Ceramide; DGTS, Diacylglyceryl-N,N,N-trimethylhomoserine; PPA, phenylpropionic acid; Glu-Gly-Arg, glutamyl-glycyl-arginine.*

*
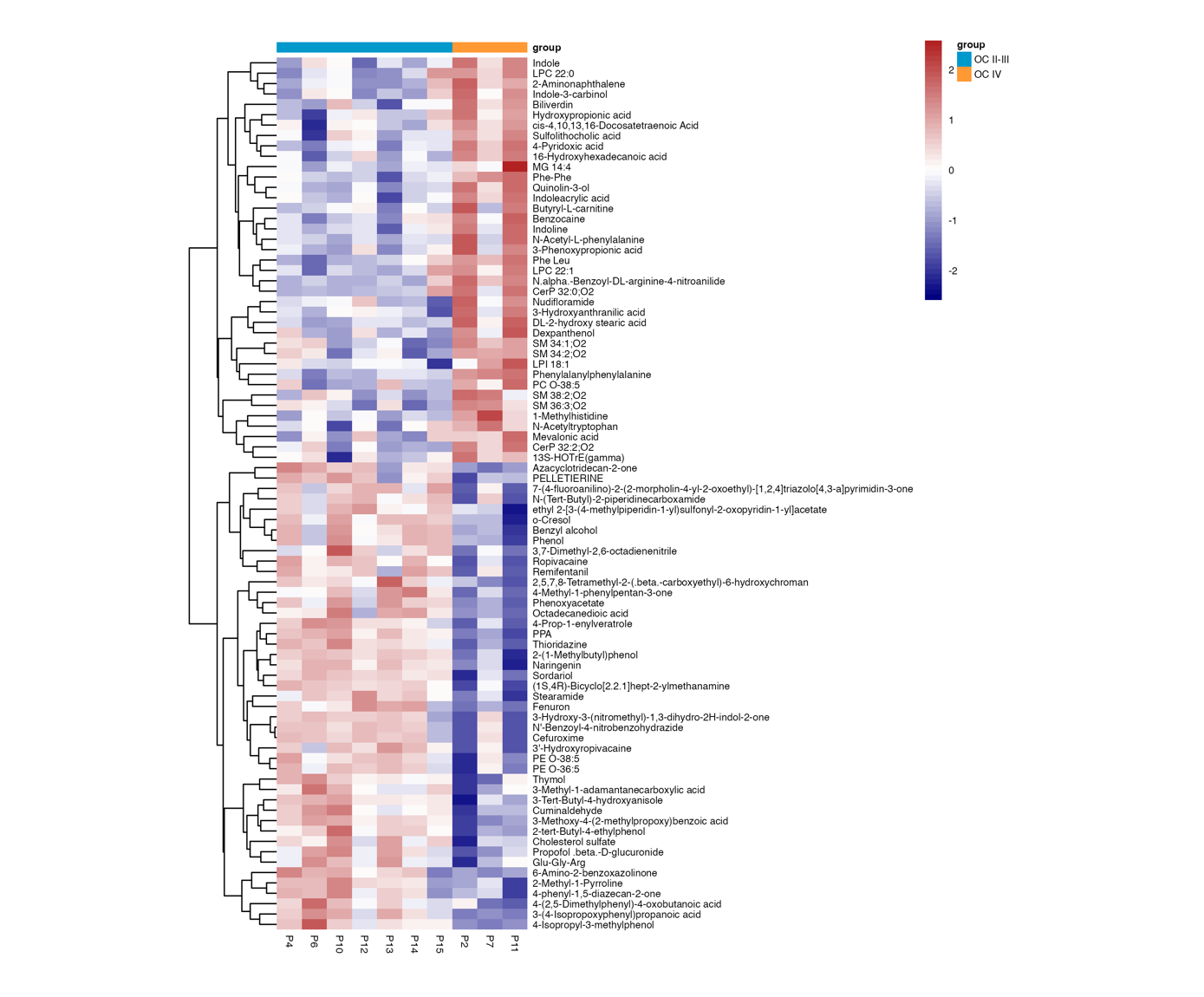
*

***Figure S2. Identification of Ascites Metabolic Signatures in OC II-III and OC IV Groups***

*The heatmap displays 84 significant metabolites (t-test, raw p-value cutoff of 0.05, fold change threshold of 1.2) across seven OC II-III and three OC IV biological replicates. Clustering was performed using Ward’s hierarchical method with Euclidean distance as the distance metric. Abbreviations: LPC, lysophosphatidylcholine; MG, Monoacylglycerol; Phe-Phe, phenylalanine- phenylalanine; Phe-Leu, phenylalanine-leucine; CerP, Ceramide phosphate; SM, sphingomyelin;* *LPI, Lysophosphatidylinositol; PC, Phosphatidylcholine; PPA, phenylpropionic acid; PE, Phosphatidylethanolamine.*


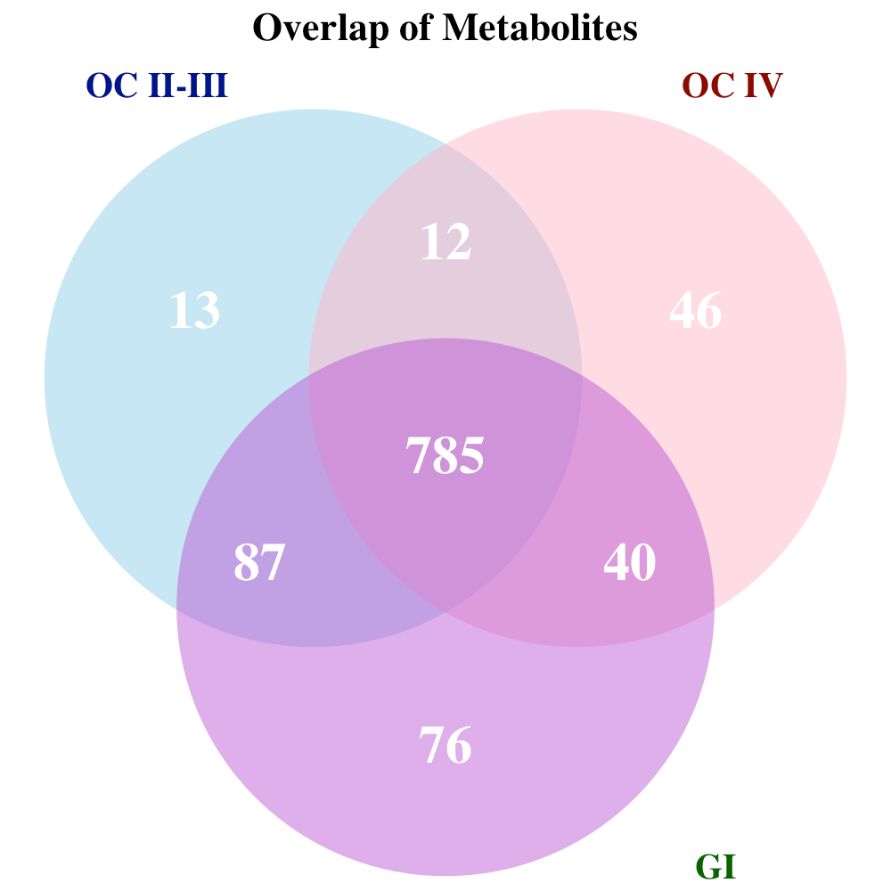


***Figure S3.*** *Venn diagram of shared vs unique metabolites across groups (OC II-III, OC IV, GI)*

**Table S9.1** Shared vs unique metabolites across groups (OC II-III, OC IV, GI)

| Only OC II-III | Only OC IV | Only GI | OC II-III & OC IV only | OC II-III & GI only | OC IV & GI only |
| --- | --- | --- | --- | --- | --- |
| 2-Hydroxy-3-isopropyl-6-methylbenzoic acid | .alpha.-Methylcinnamic acid | (+)-11-Nor-.DELTA.9-tetrahydrocannabinol-9-carboxylic acid glucuronide | 2,4,5-Trimethoxydihydrocinnamic acid | .alpha.-Hydroxymetoprolol | [8]-Dehydrogingerdione |
| 3-[(5,6-Diphenylfuro[2,3-D]pyrimidin-4-yl)amino]-1-propanol | 1,3-Bis(2-methoxyphenyl)thiourea | (2-methoxypyrimidin-5-yl)-pyrrolidin-1-ylmethanone | 4-Hydroxy-N-methylbenzamide | [3-[4-(cyclopropylmethoxymethyl)triazol-1-yl]azetidin-1-yl]-(6-methoxypyridin-3-yl)methanone | 1- (2-METHOXYPHENYL)PIERAZINE |
| Cer 28:8;O3 | 2-(4-methylanilino)pyridine-3-sulfonamide | [7-(2-Hydroxypropan-2-yl)-4a-methyl-1-methylidene-2,3,4,5,6,7,8,8a-octahydronaphthalen-2-yl] acetate | Acetylsulfamethoxazole | 1-(p-Tolyl)cyclopropanecarboxylic acid | 13-Keto-9Z,11E-octadecadienoic acid |
| Desmethyldoxepin | 2-(5-phenyl-1,2,4-oxadiazol-3-yl)ethanamine | 1,8-Diazabicyclo[5.4.0]undec-7-ene | Atorvastatin | 1,2-Bis(O-decanoyl)-sn-glyceryl-3-phosphorylcholine | 2-Phenylglycine |
| Ethoxysulfuron | 2-(Hydroxymethyl)-6-methylpyridin-3-ol | 11.alpha.-Hydroxyprogesterone .beta.-D-glucuronide | Diphenylguanidine | 1,2-Dimethylimidazole | 2-Thenoylglycine |
| Gln-Cys | 2,4-Dimethylthiazole-5-carboxylic acid | 1-Dodecanamine | Fluconazole | 1,3-Dimethyluracil | 3,19-Dihydroxyurs-12-ene-23,28-dioic acid |
| Hydroxydehydronifedipinecarboxylic acid | 2'-Aminoacetophenone | 1-Piperazineethanol, 4-dibenzo[b,f][1,4]thiazepin-11-yl- | HexCer 30:2;O2 | 11-Hydroxyundecanoic acid | 3-Cysteinylacetaminophen |
| Kaempferol | 6-(4-Methoxyphenyl)-2-sulfanylidene-2,3-dihydropyrimidin-4(1H)-one | 2-(1H-Imidazol-1-yl)-1-phenylethanone | N-Acetylsulfamethoxazole | 1-ethyl-N-methylindazole-6-carboxamide | 3-Hydroxyisovaleric acid |
| Propranolol | 7-(furan-2-carbonyl)-2-(2-methoxyethyl)-5,6,8,9-tetrahydro-[1,2,4]triazolo[4,3-d][1,4]diazepin-3-one | 2,4-Dodecadienoic acid isobutylamide | N-Methylhydantoin | 1-ethyl-N-phenylpyrazole-3-carboxamide | 4,4'-Propane-2,2-diylbis(2,6-dimethylphenol) |
| Protogenkwanin 4'-glucoside | 9-Hydroxyrisperidone | 2,5,8,11,14-Pentaoxahexadecan-16-ol | Nordazepam | 1'-Hydroxymidazolam .beta.-D-glucuronide | 4-Aminophenol |
| Pyrrocaine | AC1L6MM4 | 2-[(3-morpholin-4-yl-7-azaspiro[3.5]nonan-7-yl)methyl]benzonitrile | Tyr-Asn-Lys | 2-(hydroxymethyl)-Pyrimidine | 5-(3-fluorophenyl)-3-[[3-(4-propan-2-yloxyphenyl)-1,2,4-oxadiazol-5-yl]methyl]-3a,6a-dihydropyrrolo[3,4-d]triazole-4,6-dione |
| Thr-Ile-OH | Acetaminophen glucuronide | 2-[4-(4-methylphenyl)-9-propan-2-yl-1,5,9-triazaspiro[5.5]undec-4-en-2-yl]phenol |  | 2,3-Diaminonaphthalene | Acetoin |
| Trihexyphenidyl | Ampicillin | 2-Amino-2-methyl-4-phenylbutanoic acid | | 2-Cyano-N-(prop-2-en-1-yl)acetamide | Allocholic acid |
|  | Asn Asp Gly Val Glu | 2-ethoxy-1-[4-[4-methyl-5-(4-methylphenyl)-1,1-dioxo-1,2-thiazol-3-yl]-1,4-diazepan-1-yl]ethanone | | 2-Ethoxy-5-(1-propenyl)phenol | Arsenobetaine |
|  | Benzenesulfonic acid | 2-Methylcinchoninamide | | 3-(2-methoxyphenyl)-5,7-dimethylpyrazolo[1,5-a]pyrimidine | Atenolol |
|  | Cephalexin | 3-(2,5,7-trimethylpyrazolo[1,5-a]pyrimidin-6-yl)propanoic acid | | 3-(4-methylphenyl)-5-[2-(4-pyrimidin-2-ylpiperazin-1-yl)sulfonylphenyl]-1,2,4-oxadiazole | Cefdinir |
|  | Clopidogrel carboxylic acid | 3-(4-Phenyl-5-sulfanyl-4H-1,2,4-triazol-3-yl)-1-propanol | | 4-(3-Methyl-5-oxo-4,5-dihydro-1H-pyrazol-1-yl)benzenesulfonic acid | Cholic acid |
|  | Desaminosulfamethazine | 3,4-Dimethyl-1,2-cyclopentadione | | 4-Acetyl-N-[3-(2-amino-2-oxoethoxy)phenyl]-3-ethyl-5-methyl-1H-pyrrole-2-carboxamide | Cyclohexylsulfamate |
|  | Diatrizoic acid | 3,5-Dimethoxyphenol | | 4-Isopropyl-3-methylphenol | D-Glucurono-6,3-lactone |
|  | Ectoine | 3-Acetyl-7-diethylaminocoumarin | | 4-Methyl-N-(4-nitrophenyl)-1-piperazinecarbothioamide | Dibenzepin |
|  | ERYTHROMYCIN | 3-Hexanone | | 5-ethyl-1-(3-ethylphenyl)triazole-4-carboxylic acid | Gamma-aminobutyric acid |
|  | Eszopiclone N-oxide | 3-Methoxycatechol | | 6-Amino-2-benzoxazolinone | m-Cresol |
|  | Gemcitabine hydrochloride | 3-methyl-1-(2-phenyl-7,8-dihydro-5H-pyrido[4,3-d]pyrimidin-6-yl)butan-1-one | | 6-Benzylaminouracil | Metochlopramide |
|  | Givinostat | 4,4,6-Trimethyl-1-(4-nitrophenyl)-3,4-dihydro-2(1H)-pyrimidinethione | | 6-Hydroxy-3,4-dihydro-1(2H)-naphthalenone | Mono-2-ethylhexyl phthalate |
|  | Glutamine phenylthiohydantoin | 4-Butylmorpholine | | 7-(1H-Imidazol-1-yl)-5,6-dihydro-2-naphthalenecarboxylic acid | N-(2,6-Dimethylphenyl)-1-piperazineacetamide |
|  | L(-)-Nicotine pestanal | 4-Heptanone | | 7,7-Dimethyl-(5Z,8Z)-eicosadienoic acid | N-[3,5-bis(trifluoromethyl)phenyl]-6-oxo-1H-pyridazine-3-carboxamide |
|  | L-Histidine | 5-(2-Oxohexahydro-1H-thieno[3,4-D]imidazol-4-yl)pentanohydrazide | | 9(11)-Dehydromanogenin | N-Acetyl-DL-tryptophan |
|  | Lys Gly Ala Glu Lys | 5-Methoxytryptophan | | 9-Hydroxynonanoic acid | N-Acetyl-D-tryptophan |
|  | Meropenem | 6-Heptynoic acid | | Adenosine | N-ACETYLTRYPTAMINE |
|  | Methyl 4-[(6-deoxy-?-L-mannopyranosyl)oxy]-3,5-dimethoxybenzoate | 6-Methylquinoline | | Ala Glu Ile Lys | N-Ethyl-4-menthane-3-carboxamide |
|  | methyl N-[4-[2-(furan-2-ylmethylamino)-2-oxoethyl]-1,3-thiazol-2-yl]carbamate | 7-[[5-(4-methylphenyl)-1H-pyrazol-4-yl]methyl]-7-azaspiro[3.5]nonan-3-ol | | Antipyrine | N-tert-butyl-4-ethoxypiperidine-1-carboxamide |
|  | N,1-Diethyl-2-oxo-1,2-dihydrobenzo[cd]indole-6-sulfonamide | 9-Phenanthrol | | Azacyclotridecan-2-one | Pantoprazole |
|  | N-[(2,4-difluorophenyl)methyl]-2-(1-ethylpyrrolo[2,3-b]pyridin-3-yl)-1,3-thiazole-4-carboxamide | Acesulfame | | Azelaic acid | Pantoprazole sulfide |
|  | N-[3-(methoxymethyl)-6,7,8,9-tetrahydro-5H-[1,2,4]triazolo[4,3-a]azepin-7-yl]furan-3-carboxamide | Adenylsuccinic acid | | Benzotriazole | Paracetamol sulfate |
|  | N'-Nitrosoanabasine | Antibiotic OM 173.alpha.2 | | Carboxyibuprofen | PC 34:0 |
|  | O-Desarylranolazine | Arg Thr Ala Arg | | CATECHIN TETRAMETHYLETHER | Pipericine |
|  | PE 36:8 | Benzimidazole | | CerP 28:2;O2 | Pyridoxal |
|  | Piperacillin | Bis(2-ethylhexyl) adipate | | CerP 34:0;O2 | Risperidone |
|  | Ramipril | Cetirizine | | Cholesta-4,6-dien-3-one | S-Methyl-3-thioacetaminophen |
|  | Ranolazine dihydrochloride | Diclofenac (sodium salt) | | Cholestenone | Trigonelline |
|  | Resminostat (hydrochloride) | Didecyl hydrogen phosphate | | Cys-Trp |  |
|  | Tazobactam | Dihydromorphine | | Cytidine |  |
|  | Triethyl citrate | Diphenyl phenylphosphonate | | DG 42:7 |  |
|  | Tripropylene glycol | Enalapril |  | Diazepam | |
|  | URAPIDIL | Epi-inositol | | Diethanolamine | |
|  | Xipamide | Fenamidone | | Dodemorph | |
|  |  | Gabapentin related bis-nitrile | | Estriol |  |
|  |  | Hydromorphone | | Glycyl-L-leucine | |
|  |  | Ile Phe Gln Glu | | Gly-His |  |
|  |  | Lys Ile Gln Asp Lys | | hexadeca-9-en-1-ol | |
|  |  | Mitoxantrone | | HexCer 42:5;O3 | |
|  |  | N-(4-Acetylphenyl)-N'-(4-pyridinyl)urea | | Hexylparaben | |
|  |  | N-[(5-propan-2-yl-1,2,4-oxadiazol-3-yl)methyl]benzamide | | Homoveratric acid | |
|  |  | N-[3-(diethylamino)propyl]-2-[(4-methylphenyl)methyl]-3-oxo-1H-isoindole-1-carboxamide | | Ibuprofen .beta.-D-glucuronide | |
|  |  | N-cyclopentyl-1-(5-pyrimidin-5-yl-[1,3]thiazolo[5,4-b]pyridin-2-yl)piperidine-4-carboxamide | | Ipecac (Cephaeline) | |
|  |  | n-Octyl caffeate | | Isopentenyl adenosine | |
|  |  | Octanoic hydrazide | | Latanoprost ethylamide | |
|  |  | O-Desmethylvenlafaxine | | Leu Ile Asp Arg | |
|  |  | Olanzapine | | LPE O-14:0 | |
|  |  | Phenyl dihydrogen phosphate | | LPE O-20:2 | |
|  |  | Phenyl{[4-phenyl-6-(trifluoromethyl)-2-pyrimidinyl]sulfanyl}acetic acid | | MCI-186 |  |
|  |  | PS 44:7 |  | Metoprolol | |
|  |  | Pyridine |  | Midazolam | |
|  |  | PyroGlu-Ile-Arg | | N-(1,3,6-trimethyl-2-oxobenzimidazol-5-yl)furan-2-carboxamide | |
|  |  | Quetiapine | | N-(3-methylphenyl)-2-(4-morpholin-4-yl-1-oxophthalazin-2-yl)acetamide | |
|  |  | Quetiapine sulfoxide | | N-(4-Methoxyphenyl)-2-oxo-2H-chromene-3-carboxamide | |
|  |  | Rac-N,O-Didesmethylvenlafaxine | | N-[2-(6-oxopyridazin-1-yl)ethyl]-4-(trifluoromethoxy)benzenesulfonamide | |
|  |  | Rivaroxaban | | N-[2-(diethylamino)ethyl]-5-methyl-4-(4-methylpiperazin-1-yl)thieno[2,3-d]pyrimidine-6-carboxamide | |
|  |  | SM 30:8;O3 | | N-benzyl-5-methyl-4-oxopyrazolo[1,5-a]quinoxaline-7-carboxamide | |
|  |  | spiro[1,4-dihydroquinoxaline-3,4'-piperidine]-2-one | | N-cyclopropyl-1-oxo-3,4-dihydro-2H-pyrrolo[1,2-a]pyrazine-3-carboxamide | |
|  |  | Thr-Leu |  | N-Ethyl-2-methyl-2-propen-1-amine | |
|  |  | Tolperisone | | N-ethyl-4,6,7-trimethyl-3-oxoquinoxaline-2-carboxamide | |
|  |  | Tramadol |  | N-Methylpropionamide | |
|  |  | Tramadol HCl | | Norquetiapine | |
|  |  | Venlafaxine | | p-Acetaminobenzaldehyde | |
|  |  | Xanthene-9-carboxylic acid | | Podocarpic acid | |
|  |  |  |  | Pro Tyr Trp | |
|  |  |  |  | PS 36:2 |  |
|  |  |  |  | Psychosine | |
|  |  |  |  | SIB 1757 |  |
|  |  |  |  | SM 34:5;O2 | |
|  |  |  |  | SM 36:8;O2 | |
|  |  |  |  | SPB 16:0;O2 | |
|  |  |  |  | SPB 18:3;O2 | |
|  |  |  |  | Trans-3'-Hydroxycotinine | |
|  |  |  |  | Umbelliferone | |
|  |  |  |  | α-Hydroxymidazolam | |

**Table S9.2** Shared metabolites across groups (OC II-III, OC IV, GI)

| Shared by all (OC II-III & OC IV & GI) |
| --- |
| (.+/-.)-N,N-Dimethyl-3,4-methylenedioxyamphetamine  (+)-(S)-Carvone  (±)9-HpODE  (±)-Hexanoylcarnitine  (1R,4AR)-6-Hydroxy-1,4a-dimethyl-7-propan-2-yl-2,3,4,4b,5,6,10,10a-octahydrophenanthrene-1-carboxylic acid  (1S,4R)-Bicyclo[2.2.1]hept-2-ylmethanamine  (2E)-2,5-Dimethyl-4-vinyl-2,5-hexadien-1-yl ?-D-glucopyranoside  (2E)-4-Oxo-4-[2-(3-pyridinylcarbonyl)hydrazino]-2-butenoic acid  (2R)-3-Hydroxyisovaleroylcarnitine  (2S,4S)-1-cyclobutyl-4-phenoxypyrrolidine-2-carboxamide  (3.beta.)-Allopregnanolone sulfate  (3-Methoxy-4-hydroxyphenyl)ethylene glycol sulfate  (4-Methylphenyl)oxidanesulfonic acid  (5AR,10aR)-Octahydrodipyrrolo[1,2-a:1',2'-D]pyrazine-5,10-dione  (5E,9E)-Farnesylacetone  (9Z)-5,8,11-Trihydroxyoctadec-9-enoic acid  (9Z,12E)-15,16-Dihydroxyoctadeca-9,12-dienoic acid  (9Z,12Z)-15-Hydroxy-16-methoxyoctadeca-9,12-dienoic acid  (CIS-) NANOPHINE  (E)-2-(Hydroxymethyl)-3-(3-oxo-5-propan-2-yl-4,5,6,7-tetrahydro-1H-2-benzofuran-4-yl)prop-2-enoic acid  (E)-Ethyl 3-(2-cyanophenyl)acrylate  (R)-3-Hydroxybutyric acid  (R)-Butyrylcarnitine  .alpha.-Tocotrienol  [3-[4-(trifluoromethoxy)phenyl]-1,2,4-oxadiazol-5-yl]methyl 2,5-dimethyl-1,1-dioxo-1,2,6-thiadiazine-4-carboxylate  1-(1',3'-Benzodioxol-5'-yl)-2-butanamine  1-(1Z-Hexadecenyl)-sn-glycero-3-phosphocholine  1-(2-Hydroxyethyl)-2,2,6,6-tetramethyl-4-piperidinol  1,2-Dilinoleoylglycerol  1,3-Dimethyluric acid  1,4-Dimethyl-2,6-dioxo-1,2,5,6-tetrahydropyridine-3-carbonitrile  1.alpha.-Methyl-5.alpha.-androstan-3.alpha.-ol-17-one glucuronide  10-Hydroxy-2-decenoic acid  10-Hydroxydecanoic acid  11,14,17-Eicosatrienoic acid, (Z,Z,Z)-  11a-Hydroxyprogesterone  12(13)-Epoxy-9Z-octadecenoic acid  12-Hydroperoxy-5Z,8Z,10E,14Z,17Z-eicosapentaenoic acid  12-Hydroxydodecanoic acid  13E-Docosenamide  13-Hydroxy-9Z,11E-octadecadienoic acid  13S-HOTrE(gamma)  13S-Hydroxy-9Z,11E,15Z-octadecatrienoic acid  13Z-Docosenamide  14,15-EE-5(Z)-E  14,15-Epoxy-5Z,8Z,11Z-eicosatrienoic acid  14-Hydroxymyristic acid  15(S)-HETrE  15(S)-Hydroxy-(5Z,8Z,11Z,13E)-eicosatetraenoic acid  15S-HEPE  16,16-Dimethylprostaglandin A1  16-Hydroxyhexadecanoic acid  17.beta.-Hydroxy-17.alpha.-methyl-5.alpha.-androstan-1-en-3-one  17-Acetoxygrindelic acid  17a-Estradiol  17-Epioxandrolone  17-Hydroxy-4Z,7Z,10Z,13Z,15E,19Z-docosahexaenoic acid  17-Trifluoromethylphenyl-13,14-dihydrotrinorprostaglandin F1.alpha.  18,19-Dihydroxy-3-cleroden-15-oic acid  1-Decanoyl-2-hydroxy-sn-glycero-3-phosphocholine  1-Dodecyl-2-pyrrolidinone  1-Formylpyrrolidine-2-carboxylic acid  1-Isopropyl-3-methylbenzene  1-Lignoceroyl-2-hydroxy-sn-glycero-3-phosphocholine  1-Methyl-4-nitro-1H-imidazole  1-Methyladenosine  1-Methylguanine  1-Methylhistidine  1-O-Octadecyl-sn-glyceryl-3-phosphorylcholine  1-O-Palmitoyl-2-O-acetyl-sn-glycero-3-phosphorylcholine  1-Phenylethylamine  2-(1-Methylbutyl)phenol  2-(2-Butoxyethoxy)acetic acid  2-(4-Fluorophenyl)-N-(2-nitrobenzyl)ethanamine  2-(5-pyrazin-2-yl-1,3,4-oxadiazol-2-yl)-N-[3-(trifluoromethyl)phenyl]acetamide  2-(N-Ethyl-N-m-toluidino)ethanol  2,2'-[(4-Methylphenyl)imino]diethanol  2,2'-Methylenebis(ethyl-6-tert-butylphenol)  2,3,4,5-Tetrahydro-1H-2-benzazepine  2,4-Diisopropylphenol  2,5,7,8-Tetramethyl-2-(.beta.-carboxyethyl)-6-hydroxychroman  2,6-Dimethylpyrazine  2.alpha.-Methyl-5.alpha.-androstan-3.alpha.-ol-17-one  2-[(7-Amino-7H-[1,2,4]triazolo[4,3-b][1,2,4]triazol-3-yl)sulfanyl]-N-(2-pyrazinyl)acetamide  2-[4-(cyclopropylmethyl)-1,2,4-triazol-3-yl]-1-[(3,4-difluorophenyl)methyl]piperidine  2-[8-[cyclohexyl(methyl)amino]-3-oxo-[1,2,4]triazolo[4,3-a]pyrazin-2-yl]-N-(3-ethylphenyl)acetamide  21-hydroxy-heneicosanoic acid  2-Acetamidooctanoic acid  2-Amino-5-methylhexanoic acid  2-Aminonaphthalene  2-Cyclohexylamino-2-oxazoline  2-Ethyl-4-methyl-1H-imidazole  2-Ethylhexyl dihydrogen phosphate  2-Hydroxy-3-methylbutyric acid  2-Hydroxycaproic acid  2-Hydroxymyristic Acid  2-Hydroxypalmitic acid  2-Hydroxyphenethylamine  2-Methoxyestradiol  2-Methyl-1-Pyrroline  2-Methyl-3-ketovaleric acid  2-Methylguanosine  2-Methylindoline  2-Phenylbutyric acid  2-Pyrocatechuic acid  2-Pyrrolidinone  2-tert-Butyl-4-ethylphenol  3-(2-Methoxyphenyl)propanoic acid  3-(2-Oxocyclohexyl)propanoic acid  3-(4-Isopropoxyphenyl)propanoic acid  3-(Cyclohexylamino)-2-hydroxy-1-propanesulfonic acid  3,4,5-Trimethoxycinnamic acid  3,4-Dihydroxyhydrocinnamic acid  3,4-Dimethoxymethcathinone  3',4'-Methylenedioxy-.alpha.-pyrrolidinopropiophenone  3,5-Dimethylmorpholine  3,7-Dimethyl-2,6-octadienenitrile  3,7-Dimethyluric acid  3-[1-[6-(ethylamino)pyrimidin-4-yl]piperidin-3-yl]-N-[2-(4-methylpiperazin-1-yl)ethyl]propanamide  3-[5-(methoxymethyl)-1,2,4-oxadiazol-3-yl]-N-(2-methylpropyl)pyrrolidine-1-carboxamide  3-Amino-4-ethylbenzenesulfonic acid  3-Aminononanoic acid  3-Aminopentan-2-ol  3b-Hydroxy-5-cholenoic acid  3-Dehydroepiandrosterone sulfate  3-Dimethylaminopropionitrile  3-Hexenedioic acid  3-Hydroxy-3-(nitromethyl)-1,3-dihydro-2H-indol-2-one  3-Hydroxyanthranilic acid  3-Hydroxybutyrylcarnitine  3-Hydroxycapric acid  3-Hydroxyhexadecanoylcarnitine  3-Hydroxyoleylcarnitine  3'-Hydroxyropivacaine  3-Indolepropionic acid  3-Methoxy-4-(2-methylpropoxy)benzoic acid  3-Methoxyphenylacetic acid  3-Methyl-1-adamantanecarboxylic acid  3-Methyl-2-oxovaleric acid  3-Methylhistamine  3-Methylindole  3-Methylquinolin-4-amine  3-Methylxanthine  3-Nitrophenylhydrazine  3-Oxocholic acid  3-Phenoxypropionic acid  3-Piperidin-4-ylpropanoic acid  3-Tert-Butyl-4-hydroxyanisole  3-β-hydroxy-20-oxopregn-5-en-17-α-yl sulfate  4-(2,5-Dimethylphenyl)-4-oxobutanoic acid  4-(3,4-Dimethylphenoxy)butanoic acid  4-(Butylamino)benzoic acid  4-(Dimethylamino)-N-(1,3-thiazol-2-yl)benzamide  4-(Hydroxymethyl)benzenesulfonic acid  4,4'-(Oxydiethylene)bis(morpholine)  4-[(Isobutylamino)methyl]benzoic acid  4-[[(2S)-4,4-difluoro-2-(pyrrolidin-1-ylmethyl)pyrrolidin-1-yl]methyl]-5-(2-methoxyphenyl)-1H-pyrazole  4-Amino-2-methyl-5-pyrimidinemethanol  4-Aminoantipyrine  4-Aminohippuric acid  4-Aminomethyltetrahydropyran  4-formyl Indole  4-Hydroxy-3-(3-methylbut-2-enyl)benzoic acid  4-Hydroxybenzaldehyde  4-Ketoretinal  4-Methoxycinnamic acid  4-Methyl-1-phenylpentan-3-one  4-phenyl-1,5-diazecan-2-one  4-Pregnen-17.alpha., 20.beta.-diol-3-one-20-sulfate  4-Pregnen-6.beta.,11.beta.,17,21-tetrol-3,20-dione  4-Prop-1-enylveratrole  4-Pyridinecarboximidamide  4-Pyridoxic acid  5-(Difluoromethoxy)-2-[[(3,4-dimethoxy-2-pyridinyl)methyl]thio]-1H-benzimidazole  5(S),14(R)-Lipoxin B4  5(S),6(R)-Lipoxin A4  5,6-Dihydroxy-8Z,11Z,14Z-eicosatrienoic acid  5.alpha.-Androstan-3.beta.-ol-17-one sulfate  5.alpha.-Pregnan-3.alpha.,17-diol-20-one 3-sulfate  5-[(6,7,8-trimethoxyquinazolin-4-yl)amino]pentan-1-ol  5-Benzofurancarboxylic acid, 2,3-dihydro-2-(1-hydroxy-1,5-dimethyl-4-hexen-1-yl)-7-(3-methyl-2-buten-1-yl)-  5-Bromo-2-[(cyclopropylcarbonyl)amino]benzoic acid  5-Dodecenoic acid  5-Hydroxyindoleacetic acid  5-Hydroxytryptophol  5-Isoprostaglandin-F2.alpha.-VI  5-Isoquinolinol  5-Methyl-2-phenyl-1H-imidazole-4-carboxylic acid  5-Methylcytosine  5'-Methylthioadenosine  5-Oxo-6E,8Z,11Z,14Z-eicosatetraenoic acid  5S-Hydroxy-6E,8Z,11Z-eicosatrienoic acid  5-Tetradecynoic acid  6-(Methylamino) purine  6.beta.-Hydroxydexamethasone  6-[4-(1,3-benzodioxole-5-carbonyl)piperazin-1-yl]-2-(4-propan-2-ylphenyl)pyridazin-3-one  6-Methoxychromanone  6-Trans-12-epi-Leukotriene B4  7-(4-fluoroanilino)-2-(2-morpholin-4-yl-2-oxoethyl)-[1,2,4]triazolo[4,3-a]pyrimidin-3-one  7.alpha.,24(S)-Dihydroxy-4-cholesten-3-one  7.alpha.-Hydroxy-3-oxo-4-cholestenoic acid  7a-Hydroxy-3-oxo-5b-cholanoic acid  7-Hydroxy-8,11,13-abietatrien-19-oic acid  7-Hydroxykaurenolide  7-Hydroxymethotrexate  7-Ketocholesterol  7-Methylxanthine  7-Oxopimara-8(14),15-dien-20-oic acid  8,11,14-Eicosatrienoic acid  8,11-eicosadiynoic acid  8-Chlorotheophylline  8S,15S-Dihydroxy-5Z,9E,11Z,13E-eicosatetraenoic acid  9-(5-O-Methylpentofuranosyl)-1,9-dihydro-6H-purin-6-one  9,12-Octadecadiynoic Acid  9-Hydroxy-10E,12Z-octadecadienoic acid  9-OAHSA  9-Oxo-11-(3-pentyl-2-oxiranyl)-10E-undecenoic acid  9-OxoODE  9-OxoOTrE  9-Oxoprosta-10,12Z,14E-trienoic acid  9Z,11E,13E-Octadecatrienoic acid  AC1L7UIK  Acetaminophen  Adenine  Aldosterone  Allocystathionine  All-trans-retinoic acid  alpha-curcumene  Alpha-ketoisovaleric acid  Alpha-N-phenylacetyl-L-glutamine  Aminocaproic acid  Amoxicillin  Amprenavir  Androstan-3-ol-17-one 3-glucuronide  Androstenedione  Androsterone  Androsterone glucuronide  Angiotensin I-Converting Enzyme Substrate  Aniline  Arachidonic sulfonic acid  Arg Leu Asn Arg  Aspartame  Asymmetric dimethylarginine  Benzamide  Benzocaine  Benzoic acid  Benzyl alcohol  Betaine  Beta-Leucine  Betamethasone  Betamethasone 9,11-epoxide  Beta-N-acetylglucosamine  Bilirubin  Biliverdin  Bis(3,5,5-trimethylhexyl) phthalate  Bis(p-methylbenzylidene)sorbitol  Boldenone sulfate  Butanoic acid, 3-methyl-, 2-hydroxy-1-[hydroxy(7-methoxy-2-oxo-2H-1-benzopyran-6-yl)methyl]-2-methylpropyl ester  Butyryl-L-carnitine  Caffeine  Canthaxanthin  Capric acid  Carnosic acid  CE 18:2  CE 20:4  Cefotaxime  Cefuroxime  Cer 18:1;O2/16:0  Cer 18:2;O2/16:0  Cer 28:7;O2  Cer 32:1;O2  Cer 34:6;O3  Cer 36:2;O2  Cer 40:2;O2  Cer 42:1;O2  Cer 42:2;O2  Cer 42:3;O2  CerP 28:0;O2  CerP 28:1;O2  CerP 30:0;O2  CerP 30:1;O2  CerP 32:0;O2  CerP 32:1;O2  CerP 32:2;O2  CerP 34:1;O2  CETRIMONIUM  Cetylpyridinium  CHEBI:69439  Cholesterol sulfate  Choline  Cinnamaldehyde  Ciprostene  cis-4,10,13,16-Docosatetraenoic Acid  Cis-4,7,10,13,16,19-Docosahexaenoic acid  Cis-5,8,11,14,17-Eicosapentaenoic acid  CMPF  Coniine  Cortexolone  Corticosterone  Cortisol  Cortisol 21-sulfate  Cotinine  Creatinine  Cuminaldehyde  Cyclo(isoleucylprolyl)  Cyclo(leucylprolyl)  Cymoxanil  Cytosine  Deoxycholic acid glycine conjugate  Deoxyguanosine  Dexamethasone  Dexpanthenol  DG 34:3  DG 34:4  DG 36:3  DG 36:4  DG 36:5  DG 38:6  DG 44:8  DG O-28:2  DG O-28:5  DG O-30:2  DG O-30:4  DG O-30:5  DG O-32:3  DG O-40:8  DG O-42:7  D-Galactose  D-Glucose  D-Glutamine  DGTS 36:1  Diethyltoluamide  Diisopropanolamine  Dimethyl sulfone  Diosgenin  Diphenyl phosphate  DL-2-aminooctanoic acid  DL-2-hydroxy stearic acid  Docosahexaenoic Acid ethyl ester  Docosanamide  Dodecanedioic acid  Dodecanoic acid  Dodecylbenzenesulfonic acid  Dolastatin 10  Doxepin  Doxylamine  D-Phenyllactic acid  D-Tagatose  Erucamide  estrone 3-sulfate  Ethosuximide  ethyl 2-[3-(4-methylpiperidin-1-yl)sulfonyl-2-oxopyridin-1-yl]acetate  ethyl 4-[(Z)-(6-hydroxy-7-methyl-3-oxo-1-benzofuran-2-ylidene)methyl]piperazine-1-carboxylate  Ethyl 4-amino-2-(methylthio)-1,3-thiazole-5-carboxylate  Etofylline  Farnesyl acetone  Fenuron  Fingolimod  Foetidin  Fructose  furan-2-yl-[3-(2H-tetrazol-5-yl)piperidin-1-yl]methanone  Galactitol  gamma-Linolenoyl dopamine  Gluconic acid  Glucosamine  Glu-Gly-Arg  Glycerol tricaprylate  Glycocholic acid  Guaiacol  Guanosine  Heptadecanoic acid  Hexadecanedioic acid  Hexaethylene glycol  Hexanoyl-L-carnitine  HexCer 18:1;O2/16:0  HexCer 32:4;O3  HexCer 42:3;O2  Hippuric acid  Hydrocortisone 21-hemisuccinate  Hydroxymetronidazole  Hydroxyoctanoic acid  Hydroxypropionic acid  Hypaphorine  Hypoxanthine  Ile-Leu  Ile-Pro  Indole  Indole-3-carbinol  Indole-7-carboxaldehyde  Indoleacetaldehyde  Indoleacrylic acid  Indolelactic acid  Indoline  Indoxyl sulfate  Inosine  Isoguanine  Isoquinoline  Isovalerylglycine  KHIVORIN  Kynurenic acid  L,L-Cyclo(leucylprolyl)  L-Acetylcarnitine  L-a-Lysophosphatidylserine  L-Arachidonoylcarnitine  LARIXOL ACETATE  L-Aspartyl-L-phenylalanine  Laurylsulfuric acid  L-Carnitine  Leu Leu  Leu Leu Val Val Ala  Leucinic acid  Leu-Phe  Levofloxacin  Levoglucosan  Levulinic acid  Lidocaine  Linoleic acid  Lithocholylglycine  L-Kynurenine  L-Leucine  L-Norleucine  LPA 18:1  LPA 20:4  LPC 14:0  LPC 14:1  LPC 14:2  LPC 14:3  LPC 16:0  LPC 16:1  LPC 18:0  LPC 18:1  LPC 18:2  LPC 18:3  LPC 20:0  LPC 20:1  LPC 20:2  LPC 20:3  LPC 20:4  LPC 22:0  LPC 22:1  LPC 22:2  LPC 22:3  LPC 22:4  LPE 16:0  LPE 16:1  LPE 18:0  LPE 18:1  LPE 18:2  LPE 18:3  LPE 20:0  LPE 20:1  LPE 20:2  LPE 20:3  LPE 20:4  LPE 22:0  LPE 22:1  LPE 22:4  LPE O-16:0  LPE O-16:1  LPE O-16:2  LPE O-18:1  LPE O-18:2  LPE O-18:3  LPE O-20:1  LPG 16:0  LPG 18:1  LPG 20:4  L-Phenylalanine  L-phenylalanyl-L-proline  LPI 18:0  LPI 18:1  LPI 20:4  L-Pipecolic acid  LPS 18:0  LPS 18:1  LPS 20:0  L-Tryptophan  L-Tyrosine  Lys Val Ile Arg  m-Coumaric acid  Medroxyprogesterone  Mepivacaine  Mesoridazine  Methyl 3-(3,3-difluorocyclobutyl)-3-oxopropanoate  Methyl 6-(acetyloxy)-1,7,11-trihydroxyabieta-8,11,13-trien-18-oate  Methyl morpholine-3-carboxylate  Metoprolol acid  Metronidazole  Mevalonic acid  MG 14:2  MG 14:3  MG 14:4  MG 16:2  MG 16:3  MG 16:4  MG 18:2  MG 18:3  MG 18:4  MG 20:2  MG 20:3  MG 20:4  MG 22:3  MG 22:4  Monobutyl phthalate  Monoethylglycylxylidide (MEGX)  Monomethyl glutaric acid  Myristic acid  N-(1,5-Dimethyl-3-oxo-2-phenyl-2,3-dihydro-1H-pyrazol-4-yl)propanamide  N-(14-Methylpentadecanoyl)phenylalanine  N-(2,6-Dimethylphenyl)-1-methyl-2-piperidinecarboxamide  N-(4-ethoxyphenyl)-1-methylpiperidine-3-carboxamide  N-(4-methoxy-2-methylphenyl)-2-pyrrolidin-1-yl-5,6,7,8-tetrahydroquinazoline-6-carboxamide  N-(4-methoxyphenyl)-2-methyl-7-oxoazepane-2-carboxamide  N'-(4-Methylbenzenesulfonyl)benzohydrazide  N-(Tert-Butyl)-2-piperidinecarboxamide  N,N'-Dicyclohexylurea  N,N-Dimethylaniline  N,N-Dimethylguanosine  N.alpha.-Benzoyl-DL-arginine-4-nitroanilide  N2-(1-Oxo-4-phenylbutyl)-L-glutamine  N-Acetyl-D-galactosamine 4-sulfate  N-Acetyl-DL-valine  N-Acetyl-L-phenylalanine  N-Acetylputrescine  N-Acetylserotonin  N-Acetyltryptophan  Naringenin  N'-Benzoyl-4-nitrobenzohydrazide  N-cis-tetradec-9Z-enoyl-L-Homoserine lactone  N-Desbutylbupivacaine  Nefiracetam  Nelarabine  Neogrifolin  Neopterin  N-ethyl-1-propanoyl-2,3-dihydroindole-5-carboxamide  Niacinamide  N-Isobutyl-3-methylbutanamide  N-lactoyl-phenylalanine  N-Methylnicotinamide  N-octadecanoyl-L-Homoserine lactone  N-Oleoyl-L-Serine  NONOXYNOL-9  Norethindrone  Normetanephrine  Norsufentanil  N-Phenyldiethanolamine  N-tetradecanoyl-L-Homoserine lactone  Nudifloramide  Nutriacholic acid  o-Cresol  Octadecanedioic acid  Octanoylcarnitine  Octodrine  Oleamide  Oleoyl ethylamide  Olivetol  Ortho-hydroxyphenylacetic acid  o-Tyrosine  Oxindole  o-Xylene  Oxypurinol  Palmitic amide  Palmitoyl-L-carnitine  p-Anisic acid  Panthenol  Pantothenic acid  PC 28:0  PC 30:0  PC 32:1  PC 32:2  PC 34:1  PC 34:2  PC 34:3  PC 36:3  PC 36:4  PC 36:5  PC 38:3  PC 38:4  PC 38:6  PC 38:7  PC 40:4  PC 40:6  PC O-28:7  PC O-32:0  PC O-34:1  PC O-34:2  PC O-36:3  PC O-36:4  PC O-36:5  PC O-36:6  PC O-38:4  PC O-38:5  PC O-38:6  PC O-40:5  PC O-40:6  PC(16:0/16:0)  PC(18:1(9Z)/18:1(9Z))  PE 30:3  PE 30:7  PE 34:0  PE 34:2  PE 36:1  PE 36:2  PE 36:4  PE 36:5  PE 38:1  PE 38:2  PE 38:3  PE 38:4  PE 38:5  PE 38:6  PE 40:4  PE 40:6  PE O-34:3  PE O-36:5  PE O-36:6  PE O-38:5  PE O-38:6  PE O-38:7  PE O-40:7  PE O-40:8  PELLETIERINE  Pentaethylene glycol  Perfluorooctanoic acid  PFNA  PG 18:1_18:2  PGPC  Phe Leu  Phe Val  Phe-Leu  Phenol  Phenoxyacetate  Phenylacetaldehyde  Phenylalanylphenylalanine  Phenylpropanolamine  Phe-Phe  Phe-Pro  Phe-Trp  Phosphocholine  Phthalic acid  Phthalic anhydride  Phthalide  PI 38:4  PI 38:5  Piperazine-2-carboxylic acid  Piperine  PPA  Prednisolone 21-sulfate  Prednisone  Pregnenolone sulfate  Pro Ile  Pro-Leu  Propofol .beta.-D-glucuronide  Prostaglandin H2  Prostaglandin I2  PS 36:0  PS 36:1  PS 38:2  PS 38:3  PS 40:5  p-Synephrine  p-Tolyl Sulfate  Pyridoxamine  Pyroglutamic acid  PyroGlu-Val  Quinolin-2-ol  Quinolin-3-ol  Quinolin-8-ol  Ranolazine  Remifentanil  Resveratrol-3-O-sulfate  Riboflavin  Ribothymidine  Ricinoleic acid  Rivastigmine  Ropivacaine  Salicylic acid  Salicyluric acid  Sepiapterin  SM 18:1;O2/12:0  SM 18:1;O2/16:0  SM 18:2;O2/16:0  SM 18:2;O2/16:1  SM 28:1;O2  SM 28:2;O2  SM 28:8;O2  SM 30:1;O2  SM 30:2;O2  SM 32:1;O2  SM 32:2;O2  SM 34:1;O2  SM 34:1;O3  SM 34:2;O2  SM 34:2;O3  SM 34:4;O2  SM 36:2;O2  SM 36:3;O2  SM 38:1;O2  SM 38:2;O2  SM 38:3;O2  SM 38:4;O2  SM 40:3;O2  SM 40:4;O2  SM 40:7;O2  SM 40:8;O2  SM 42:4;O3  SM 42:5;O2  SM 42:6;O2  SM 44:6;O2  Sorbitol  Sordariol  SPB 16:0;O3  SPB 18:0;O3  SPB 18:1;O3  SPB 18:4;O2  SPB 20:0;O2  SPB 20:1;O2  SPB 20:1;O3  SPB 20:2;O2  SPB 22:1;O2  SPB 22:2;O2  SPB 22:3;O2  Spermine  Sphinganine  Sphingosine  SPHINGOSYLPHOSPHORYL CHOLINE  SQDG 28:6  Stachydrine  Stearamide  Stearic acid  Stearic Acid ethyl ester  Styrene  Sufentanyl  Sulfamethoxazole  Sulfolithocholic acid  Taurine  Taurocholic acid  Taurodeoxycholic acid  Terephthalic acid  Tetradecanedioic acid  Tetraethylene glycol  Tetrahydrocortisone  TG 46:1  TG 46:2  TG 46:3  TG 46:5  TG 48:3  TG 48:4  TG 48:6  Theobromine  Theophylline  Thiamine  Thioridazine  Threonic acid  Thr-Val-Leu  Thymol  Torasemide  Torsemide  Trans-Cinnamic acid  Tranylcypromine hydrochloride  Triethanolamine  Triisopropanolamine  Trimethoprim  Trimethylamine  Trimethylamine N-oxide  Triphenylphosphine oxide  Tris(2-ethylhexyl) trimellitate  Tris(butoxyethyl)phosphate  Tropine  Trp Leu  Tryptamine  Tyr Glu Lys Thr Tyr  Tyr-Leu  Tyr-Phe  U-44069  Ureidopropionic acid  Uric acid  Uridine  Urocanic acid  Ursodeoxycholic acid  Vaccenic acid  Z-Ligustilide  γ-CEHC  Δ2-cis-Hexadecenoic Acid  δ-Valerolactam |
